# Reliable Single Cell Array CGH for Clinical Samples

**DOI:** 10.1371/journal.pone.0085907

**Published:** 2014-01-21

**Authors:** Zbigniew T. Czyż, Martin Hoffmann, Günter Schlimok, Bernhard Polzer, Christoph A. Klein

**Affiliations:** 1 Experimental Medicine and Therapy Research, University of Regensburg, Regensburg, Germany; 2 Project Group Personalized Tumor Therapy, Fraunhofer Institute for Toxicology and Experimental Medicine ITEM, Regensburg, Germany; 3 Hospital Augsburg, II. Medical Center, Augsburg, Germany; The Chinese University of Hong Kong, Hong Kong

## Abstract

**Background:**

Disseminated cancer cells (DCCs) and circulating tumor cells (CTCs) are extremely rare, but comprise the precursors cells of distant metastases or therapy resistant cells. The detailed molecular analysis of these cells may help to identify key events of cancer cell dissemination, metastatic colony formation and systemic therapy escape.

**Methodology/Principal Findings:**

Using the *Ampli1™* whole genome amplification (WGA) technology and high-resolution oligonucleotide aCGH microarrays we optimized conditions for the analysis of structural copy number changes. The protocol presented here enables reliable detection of numerical genomic alterations as small as 0.1 Mb in a single cell. Analysis of single cells from well-characterized cell lines and single normal cells confirmed the stringent quantitative nature of the amplification and hybridization protocol. Importantly, fixation and staining procedures used to detect DCCs showed no significant impact on the outcome of the analysis, proving the clinical usability of our method. In a proof-of-principle study we tracked the chromosomal changes of single DCCs over a full course of high-dose chemotherapy treatment by isolating and analyzing DCCs of an individual breast cancer patient at four different time points.

**Conclusions/Significance:**

The protocol enables detailed genome analysis of DCCs and thereby assessment of the clonal evolution during the natural course of the disease and under selection pressures. The results from an exemplary patient provide evidence that DCCs surviving selective therapeutic conditions may be recruited from a pool of genomically less advanced cells, which display a stable subset of specific genomic alterations.

## Introduction

Comprehensive analysis of minute quantities of genomic DNA has become important in a variety of forensic, diagnostic and biological studies. For example, in cancer research or pre-implantation diagnostics, the number of available cells for downstream analyses may be as low as one single cell. In cancer research, single-cell technologies are increasingly needed to study the course of metastatic spread of cancer cells. Multiple studies conducted in the past have shown that the presence of circulating tumor cells (CTCs) in the peripheral blood or disseminated cancer cells (DCCs) in the bone marrow (BM) or lymph nodes (LN) is an independent prognostic factor of poor outcome of almost all tested cancer types [1]–[5]. Strikingly, it could be shown that cancer cells disseminate very early during the course of disease and evolve in parallel to the tumor cells at the primary site [6]–[8]. These findings were supported by significant genetic disparity observed between the primary tumors (PTs) and corresponding DCCs [9]–[11] as well as among DCCs themselves [12]. Subsequent functional studies demonstrated that, at least in the case of esophageal cancer, DCCs show different susceptibility to applied anti-cancer treatment than cancer cells originating from the primary lesion [11]. In line with this, studies in breast cancer have shown that DCCs and CTCs may survive the first line treatment indicating their intrinsic or acquired resistance to cancer therapy [13], [14]. For all of these reasons, detailed analysis of DCCs and CTCs may help to identify genes and pathways allowing cancer cells to leave the primary lesion, survive in the circulation for extended periods of time, colonize distant sites, and survive systemic therapies.

A variety of analytical techniques have been developed to amplify and study the genomes of single-cells [15]–[23]. Chromosomal comparative genomic hybridization (cCGH) could be adapted to analyze single-cell DNA and identify highly penetrant alterations in the genomes of DCCs [19]. This method, although comprehensive, is very labor-intensive and allows only detection of aberrant regions larger than 10–20 Mb. Implementation of array CGH (aCGH) technology revolutionized the study of single-cell cancer genomes. A single-cell aCGH assay using tiling path BAC array platform described by Fiegler et al. allowed detection of a deletion of 8.3 Mb [24]. Using arrays composed of highly purified BAC clones previously we identified aberrant regions as small as 1–2 Mb in cell lines and 4.8 Mb in DCCs [25]. More recent studies indicate that using high-density oligonucleotide microarrays the detection limit of single-cell aCGH can be reduced to 1 Mb or less in freshly isolated cells [26], [27]. Despite these advances an additional hurdle consists in the requirements imposed by clinical samples. So far, it has not been extensively studied how fixation and staining methods used to identify CTCs and DCCs may influence the outcome of the single-cell aCGH.

The objective here was to establish a robust single-cell aCGH protocol allowing reliable detection of genomic alterations in patient-derived DCCs. We applied *Ampli1™* single-cell WGA technology together with SuperPrint G3 4×180 k Agilent aCGH microarrays to provide a precise and easy to use workflow for high-resolution assessment of copy number changes in single cells. We show that the new workflow displays high specificity and enables reliable assessment of the copy number changes in single DCCs, which may be used to address the cellular heterogeneity in cancer. Finally, we demonstrate the potential of our new technique in a case study of DCCs isolated from a patient with advanced breast cancer disease during the course of high-dose chemotherapy treatment.

## Materials and Methods

### Ethics statement

Bone marrow sampling was performed within the study protocol of the GEBDIS study at the Central Hospital in Augsburg after informed written consent of patients was obtained. The ethics committees of the University of Tübingen and of the University of Regensburg (ethics vote number 07-079) approved bone marrow sampling and genomic analysis of the isolated cells. Additionally, as control and reference samples, we used single cells from the mononuclear cell fraction of peripheral blood obtained from five healthy donors. Three donors provided written informed consent after obtaining approval by the ethics committee of the University of Regensburg (ethics vote number 12-101-0038) and two healthy donors provided verbal informed consent. The latter samples were taken before 2008 when no ethics vote for voluntary blood donations of healthy donors was required.

### Cell lines

BT-474 and SKBR3 cell lines were obtained from repository of the German Collection of Microorganisms and Cell Cultures at the Leibniz Institute DSMZ (Braunschweig, Germany), other cell lines including MDA-MB-453 and MDA-MB-361 were obtained from the American Type Culture Collection (ATCC). OE-19 was obtained from the European Collection of Cell Cultures. PT1590 cell line was originally generated at the University Medical Center Hamburg-Eppendorf [28]. All cell lines have been maintained in the conditions recommended by the distributor. Identity of all cell lines was confirmed using a PCR-based fingerprinting.

### Detection and isolation of disseminated cancer cells

The procedure for bone marrow preparation has been described elsewhere [12], [29]. Bone marrow or blood cells (1–2×10^6^) were stained using either monoclonal antibody clone A45-B/B3 against cytokeratins 8,18,19 or monoclonal antibody clone CK2 against cytokeratin 18. Control cells (from healthy donors) were stained with monoclonal antibody specific for vimentin (clone V9, Dako). Mouse IgG1 Kappa (MOPC-21) was used as isotype control for all immunocytochemical experiments. Visualization was carried out using alkaline phosphatase/anti-alkaline phosphatase technique with 5-bromo-4-chloro-3-indolyl phosphate and nitroblue tetrazolium (BCIP/NBT) used as substrate.

### Primary whole genome amplification (WGA)

All single cells, cell pools (few hundred cells) and microdissected specimens from formalin fixed paraffin embedded (FFPE) tumor tissue were amplified using the *Ampli1*™ WGA Kit (Silicon Biosystems) [12], [19].

### Re-amplification of the WGA products

Re-amplification was performed in a volume of 50 µl. Each PCR reaction was composed of the following ingredients: 5 µl Expand Long Template Buffer 1 (Roche Diagnostic), 1 µM of the LIB1 (5′-TAGTGGGATTCCTGCTGTCAGT-3′) or MseLig-21 primer (5′-AGTGGGATTCCTGCTGTCAGT-3′) – depending on the adapter used in the primary WGA, 1.75 µl dNTPs (10 mM), 1.25 µl BSA (Roche Diagnostic), 2.5 U of Expand-Long-Template DNA Polymerase (Roche Diagnostic) and 1.0 µl of the template DNA. The MJ thermocycler was set as follows: 1 cycle of 94°C for 60 sec, 60°C for 30 sec, 65°C for 2 min, 10 cycles of 94°C for 30 sec, 60°C for 30 sec, 65°C for 2 min (extended by 20 sec/cycle). Typically three reactions were run in parallel, which were pooled and used as template for DNA labeling and aCGH. A negative control was included in every run.

### Labeling of sample DNA

**Random-primed DNA labeling approach (RP labeling).** Test and reference DNA samples were labeled using SureTag DNA Labeling Kit (Agilent Technologies) according to the instruction provided by the supplier (Agilent Oligonucleotide Array-Based CGH for Genomic DNA Analysis, version 7.1, December 2011). Briefly, 1.5 – 2.0 µg of the purified input DNA (WGA product or unamplified genomic DNA) was supplemented with 5 µl of Random Primer Mix and filled up with H_2_O to 31 µl. Unamplified DNA and WGA products samples were denatured at 95°C for 10 or 3 minutes, respectively. Sample tubes were transferred on ice and incubated for 5 min. The labeling reaction with exo-Klenow fragment consisted of the following ingredients: 31 µl of denatured DNA, 10 µl of 5x Reaction Buffer, 5.0 µl of 10x dNTP Mix, 3.0 µl of Cy5-dUTP (test) or Cy3-dUTP (reference) and 1.0 µl of Exo(–) Klenow fragment. Labeling reaction was run at 37°C for two hours, followed by an inactivation step at 65°C for 10 minutes. Labeled DNA was purified using Ultra 0.5 purification system with a size cut-off of 30 kDa. DNA yields and dye incorporation rates were quantified using the NanoDrop ND-1000 instrument.

**PCR-based labeling.** *PCR-based labeling using dye-conjugated universal primer (PCR-T1).* Placement of the dye on the universal primer provides the advantage that all restriction digestion fragments present in the WGA product irrespectively of their size will be labeled with the same amount of dye. To avoid cross-hybridization of adapter sequences flanking amplicons in the WGA products, test and reference samples were labeled using different PCR-adapters. Test samples were labeled with the PCR-adapter incorporated in the *Ampli1*™ WGA kit, while all the reference DNA samples were amplified using the following adapters: MIB5 (5′-TGAGCTGGTCATTGCGCATGGT-3′) and ddMse XI (5′-TAACCATGCGC-3′). Universal primers used in the labeling reaction were directly conjugated with either Cy5 in the case of *Ampli1™* universal primer [5′-TAGTGGGATTCCTGCTGTCAGT-3′] or Cy3 in the cases of MIB5 primer [5′-TGAGCTGGTCATTGCGCATGGT-3′] (underscores indicate the placement of the dye). The labeling PCR was run in a total volume of 50 µl reaction composed of 5 µl of 10x Expanded Long Template Buffer 1 (Roche Diagnostics), 2.4 µM of dye-conjugated LIB1 or MIB5 primer (for test and reference sample, respectively), 350 µM of dNTPs, 0.5 µl BSA (Roche), 3.75 U of Expand-Long-Template DNA Polymerase (Roche Diagnostics) and 1.0 µl of the template DNA. The PCR was programmed as follows: 10 cycle of 94°C for 15 sec, 51°C for 30 sec and 65°C for 3∶30 min, 2 cycles of 94°C for 15 sec, 51°C for 30 sec and 65°C for 3:30 min (extended by 10 sec every cycle), followed by a final elongation step of 7 min at 65°C. Products were purified using the Amicon Ultra 0.5 System (cut-off size 100 kDa) and quantified with the NanoDrop ND-1000 instrument.

*PCR-based labeling using incorporation of dye-conjugated dNTPs (PCR-T2).* WGA products were labeled by PCR in the presence of Cy5 or Cy3 conjugated dCTP and dUTP. Incorporation of the fluorescent dyes using the dNTPs provided the advantage that adapters flanking each amplicon in the WGA product could be removed prior to aCGH hybridization, thereby avoiding unspecific cross-hybridization of test sample with reference DNA and oligonucleotide probes on the array. For each sample two 50µl PCR reactions were run in parallel each comprising 5 µl of 10x Expanded Long Template Buffer 1 (Roche Diagnostics), 2.4 µM of the universal primer (LIB1 or MseLig-21, depending on the adapter sequence incorporated in the WGA product), 350 µM of dATP and dGTP, 315 µM of dCTP and dTTP, 35 µM of Cy5/Cy3-dCTP and Cy5/Cy3-dUTP (GE Healthcare), 0.5 µl of BSA (Roche Diagnostics), 7.5 U of Expand-Long-Template DNA Polymerase (Roche) and 0.5 µl of the template DNA. The PCR cycler was programmed as follows: 10 cycle of 94°C for 15 sec, 60°C for 30 sec and 65°C for 3:30 min, 2 cycles of 94°C for 15 sec, 60°C for 30 sec and 65°C for 3:30 min (extended by 10 sec every cycle), followed by a final elongation step of 7 min at 65°C. Subsequently, the resulting PCR products were subjected to digestion with Tru1I restriction endonuclease to cleave off the PCR-adapters. For this purpose the PCR product was supplemented with 5.6µl of Buffer R and 30 U of Tru1I. Digestion was performed at 65°C for 3 hours. Resulting products were pooled and purified using the Amicon Ultra 0.5 System (100 kDa cut-off). DNA yields and dye incorporation rates were quantified using the NanoDrop ND-1000 Instrument.

### Array comparative genomic hybridization

Array CGH was performed on oligonucleotide-based SurePrint G3 Human CGH 4×180K microarray slides (design code: 022060) according the protocol provided by the manufacturer (Agilent Oligonucleotide Array-Based CGH for Genomic DNA Analysis, version 7.1, December 2011). Slight modifications were introduced WGA products processed with the PCR-based labeling approaches. Here, the hybridization mix consisted of 5.0 µg of Cot1-DNA (Roche Diagnostics), 12 µl of 10x Blocking Reagent (Agilent Technologies), 60 µl of 2x Hi RPM Hybridization Buffer, 1% (v/v) of both Tween20 and Igepal and 19 µl of both test and reference DNA. For each hybridization 100 µl of the hybridization mix was applied on the array and hybridized at 65°C for 24 h. Following the hybridization, the slides were washed twice for 2:30 min in Oligo aCGH/ChIP-on-Chip Wash Buffer 1 (Agilent Technologies) at room temperature, twice for 30 sec min in Oligo aCGH/ChIP-on-Chip Wash Buffer 2 (Agilent Technologies) at 37°C. Washed slides were immersed in acetonitrile to remove all remaining traces of the wash buffers. Finally, slides were scanned using an Agilent Microarray Scanner Type C.

### Processing and analysis of the aCGH data

Microarray TIFF image files were processed with the Agilent Genomic Feature Extraction Software (version 10.7). The resulting text files were imported and analyzed with Agilent Genomic Workbench Software (version 6.5 Lite). Aberrant regions were recognized using ADM-2 algorithm with threshold set to 6.5. Centralization algorithm was set to a threshold of 6.0 and bin size of 10. To avoid false positive calls, aberration filters were applied to define the minimum log2 ratio (0.25 for unamplified DNA or WGA products from freshly picked cell and 0.3 for WGA product from cell stained for vimentin or cytokeratins) and the minimum number of probes in an aberrant interval (5 and 25 for unamplified DNA and WGA products, respectively). The microarray data presented in this manuscript has been deposited at Gene Expression Omnibus (GEO) database (accession number: GSE52366).

### Identification of minimal regions of aberration (MRAs)

MRA refers to the smallest region of genomic copy number alteration overlapping across an analyzed set of aCGH profiles.

### Statistical analysis

Identification accuracy was assessed using separate ROC curves for gains and losses. To this end segmentation profiles obtained by the ADM2 algorithm were binarized according to threshold values (Thr): for gains, values ≥ Thr were set to unity and 0 otherwise, for losses: values ≤ –Thr were set to unity and 0 otherwise. Reference profiles were binarized according to a fixed threshold of Thr  =  0.25, while test profiles were binarized for different values of Thr. ROC curves were obtained by comparing a reference binarization to test binarizations for different positive threshold values. For clustering, segmentation profiles were discretized according three levels, i.e. values ≥ Thr were labeled as gains by 1, values ≤ –Thr were labeled as losses by –1, and all other values were labeled by zero. Hierarchical clustering was performed using Euclidean distance and agglomeration by complete linkage. Analyses were done using R [R: A Language and Environment for Statistical Computing, R Core Team,R Foundation for Statistical Computing, Vienna, Austria, 2013, url  =  http://www.R-project.org].

## Results

**Figure 1:**
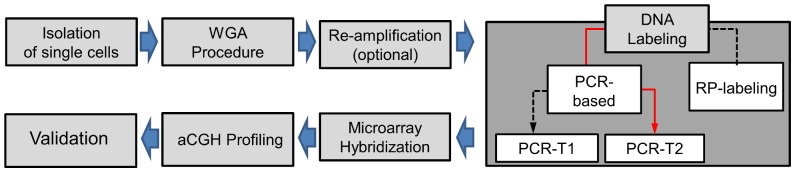
Experimental approach. Overview of the single cell aCGH procedure: single cells were isolated by micromanipulation and subjected to *Ampli1™* WGA protocol. Primary WGA product can be re-amplified for further downstream applications (optional step). DNA was labeled by RP labeling or PCR-based approaches (PCR-T1 and PCR-T2). Subsequent hybridization was carried out on Agilent SurePrint G3 Human 4×180k arrays resulting data was evaluated with Agilent Genomic Workbench Software.

Figure 1 provides an overview of the sample processing and the experimental set-up used to obtain optimized assessment of single cell copy number changes. Based on our experience with single cell cCGH we focused on those labeling methods that are compatible with the WGA procedure and may have an impact on the aberration calls.

### Optimization and validation of the single-cell aCGH assay

First, we developed two customized PCR-based approaches for labeling *Ampli1™* single-cell WGA products and compared them with the recently published protocol using random-primed isothermal incorporation of fluorescent dyes (RP labeling) [27]. The first PCR-based protocol employed a dye-conjugated universal primer (PCR-T1) that binds to the adapter sequence utilized in the primary WGA. The second approach was based on the incorporation of dye-conjugated dNTPs (PCR-T2) during a short re-amplification step. Both techniques utilized the same universal primer as incorporated in the *Ampli1™* WGA procedure, thereby enabling comprehensive labeling of the complete representation of single-cell DNA. Both labeling approaches were tested for their accuracy and reliability using PT1590 esophageal cancer cell line cells [28]. We generated three single-cell and two WGA products of cells pooled from PT1590 cell line. Paired samples (single cell and cell pool) for both PT1590 cell line and healthy donor were labeled in parallel with both techniques and subsequently hybridized on Agilent SurePrint G3 Human 4×180k arrays. As benchmark, we used aCGH profiles obtained with unamplified genomic DNA. Samples processed with PCR-T1 provided consistently higher dye incorporation rates, whereas PCR-T2 generated higher DNA yields (Table S1 in the Text S1). More importantly, signal intensities and signal-to-noise ratios obtained for both dyes (Cy5-red/test and Cy3-green/reference), were higher for samples processed with PCR-T2. The direct comparison of PCR-T1 and PCR-T2 revealed striking disparities between single-cell aCGH profiles of the same individual cell (Figure S1A-C). When compared to bulk DNA we found that multiple genomic alterations could be detected only if labeling PCR-T2 was applied (Figure S1A-C). The better fit of PCR-T2 aberrations with results from bulk genomic DNA was quantified by subsequent ROC-analysis (Figure S1D). In addition, repeated hybridization of single-cell WGA products processed with PCR-T2 gave highly reproducible results (Figure S2).

To further improve the performance of the assay we searched for the best performing reference sample. To this end we compared two sample types: (i) WGA products generated from cell pools and (ii) samples obtained by pooling four single-cell WGA products. Direct comparison revealed that derivative log2 ratio spread (DLRS) values, a measurement of hybridization noise, were consistently higher when WGA products from cell pools were used as reference (Figure S2). As a consequence all subsequent experiments included a pool of four single cell WGA products originating from one healthy individual as a reference sample.

**Figure 2:**
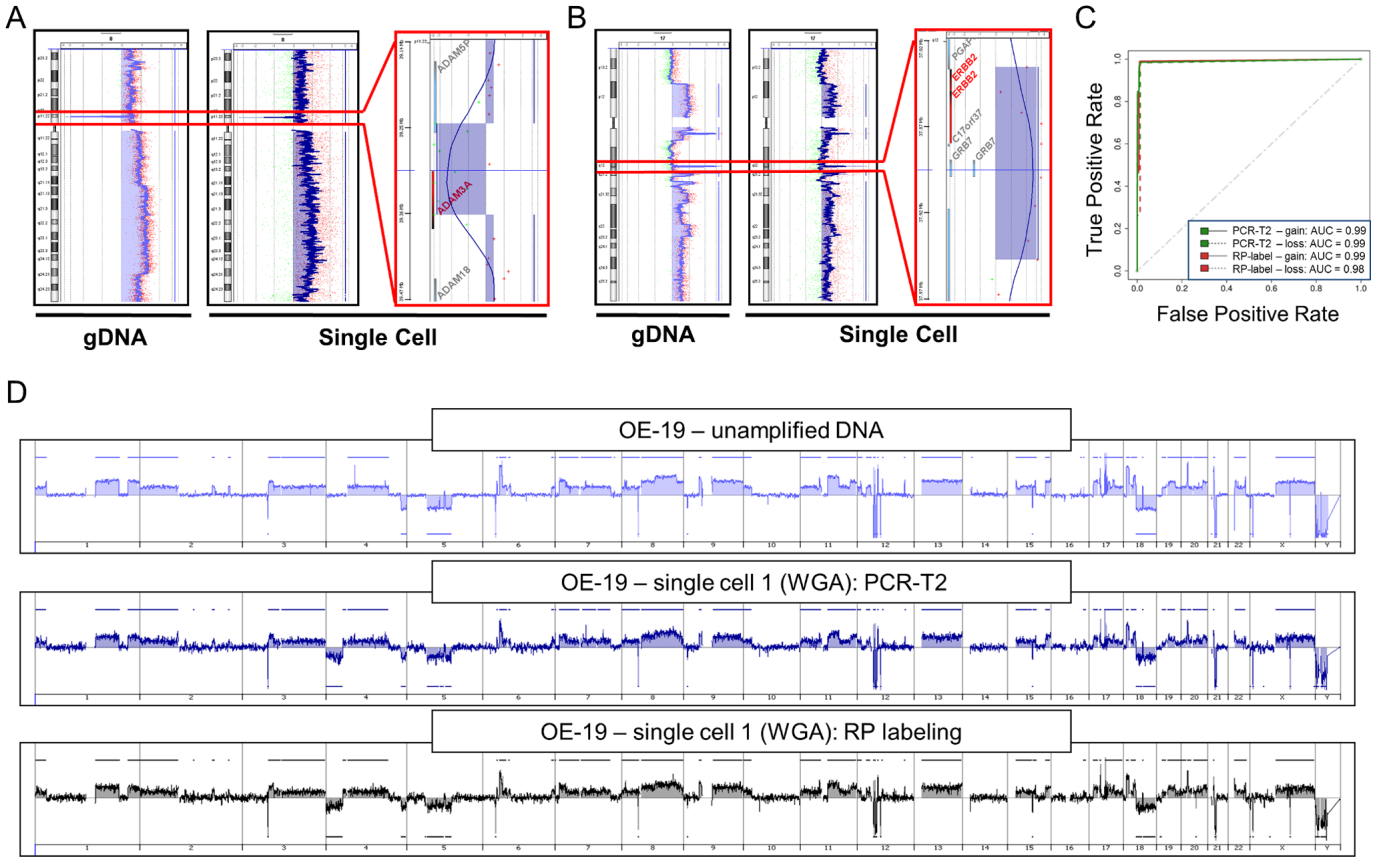
PCR-based PCR-T2 labeling technique vs. RP labeling. A) Chromosome specific aCGH profiles of chromosome 8. B) Chromosome specific aCGH profiles of 17. Each panel represents aCGH profiles generated with unamplified and single-cell gDNA (PCR-T2 labeling) – left and middle plot, respectively. The right plot of each panel represents magnified graphical overview of genes within loci recognized as aberrant. C) ROC-curves (corresponding to profiles presented in panel A) depicting the accuracy of single cell aCGH assay for PCR-T2 or RP labeling. The array CGH profile generated using unamplified gDNA of OE-19 cells was taken as reference for the comparison. ROC analysis was performed on a genome-wide basis. D) Genome wide aCGH profiles of OE-19 cells generated using unamplified gDNA (upper panel) and a single-cell WGA product labeled with PCR-T2 (middle panel) or RP labeling approach.

Next we compared the performance of PCR-T2 with the recently published RP labeling procedure [27]. We started with using freshly picked single cells of OE-19 esophageal cancer cells. Irrespectively of the labeling approach used, two aberrations of only 0.1 Mb in size (one homozygous deletion and one amplification) could be reliably and reproducibly detected in all OE-19 single cells (Figure 2A-B) and the aCGH profiles of single OE-19 cells were highly concordant with the results of unamplified DNA (Figure 2C-D). In addition, we tested both labeling techniques when applied on re-amplified WGA products of OE-19 cells. Independently of the labeling technique applied, aCGH profiles of both re-amplified and primary single-cell WGA products as well as with unamplified DNA showed high level of concordance (Figure S3A-B). This was also true for single cell WGA products obtained from healthy donors displaying balanced profiles (Figure S3C). Therefore, the second round of amplification did not introduce a significant amplification bias. Since fixation and staining protocols used for detection of DCCs may affect the quality of single cell DNA [30], [31], we also compared PCR-T2 and the RP labeling for clinical samples. Here, we could show that when applied to immunostained cells PCR-T2 is more robust and less susceptible to generate technical bias than it is the case with RP labeling. Therefore, PCR-T2 became the method of choice for processing the *Ampli1™* WGA product in all subsequent experiments (Figure S6 and Figure S7; text S1).

### Detection of cell-to-cell heterogeneity in single cells

**Figure 3:**
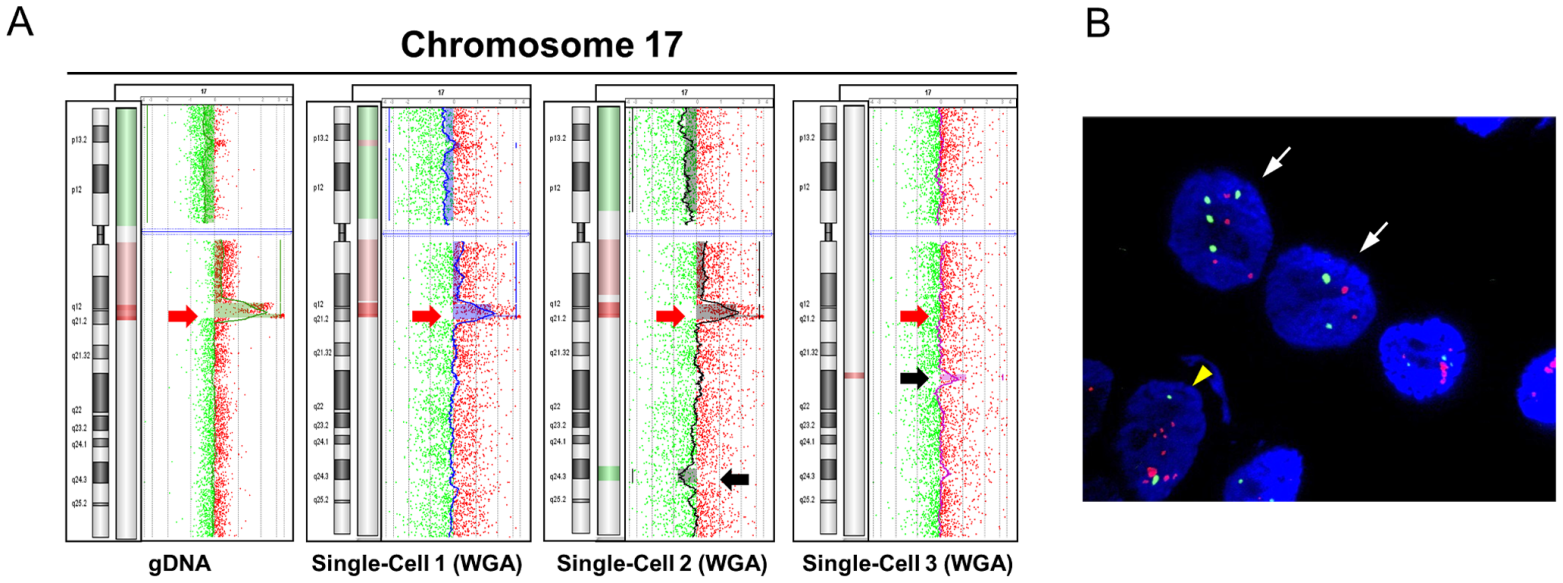
Assessment of the cellular heterogeneity. A) Vertical aCGH profiles of chromosome 17 for unamplified gDNA and single-cell WGA products of PT1590 cells. Red arrow indicate ERBB2 locus. Note, one single cell (cell 3) shows a balanced profile at this site. Black arrows indicate private genetic alteration detected only in individual cells. B) Representative FISH images of PT1590 cells. Red signals indicate ERBB2 locus and green CEP17. White arrows label cells with balanced copy number of ERBB2 vs. CEP17. Yellow arrowhead shows cells with high-level amplification of the ERBB2 locus.

We evaluated the capability of our assay based on PCR-T2 labeling to assess genomic heterogeneity at the single cell level. We analyzed three single-cell WGA products, cell-pool WGA product and corresponding unamplified DNA of PT-1590 cell line. PT-1590 cells were previously shown to harbor amplification of ERBB2 gene [11]. Surprisingly, we found by aCGH analysis that one out of three analyzed PT1590 cells did not display the amplification of ERBB2 (Figure 3, Figure S4). To determine whether this finding was a technical artifact or reflecting true cellular heterogeneity, we performed FISH analysis. We found that about 10% of the PT1590 cells indeed displayed a balanced copy number ratio at the ERBB2 locus (Figure 3B). CNAs found in single cells only may thus represent true-positive events that escape detection in the bulk DNA where they are masked by the heterogeneity of the cell population. (Figure 3B, Figure S4).

### Quantitative assessment of CNAs in single cells

**Figure 4:**
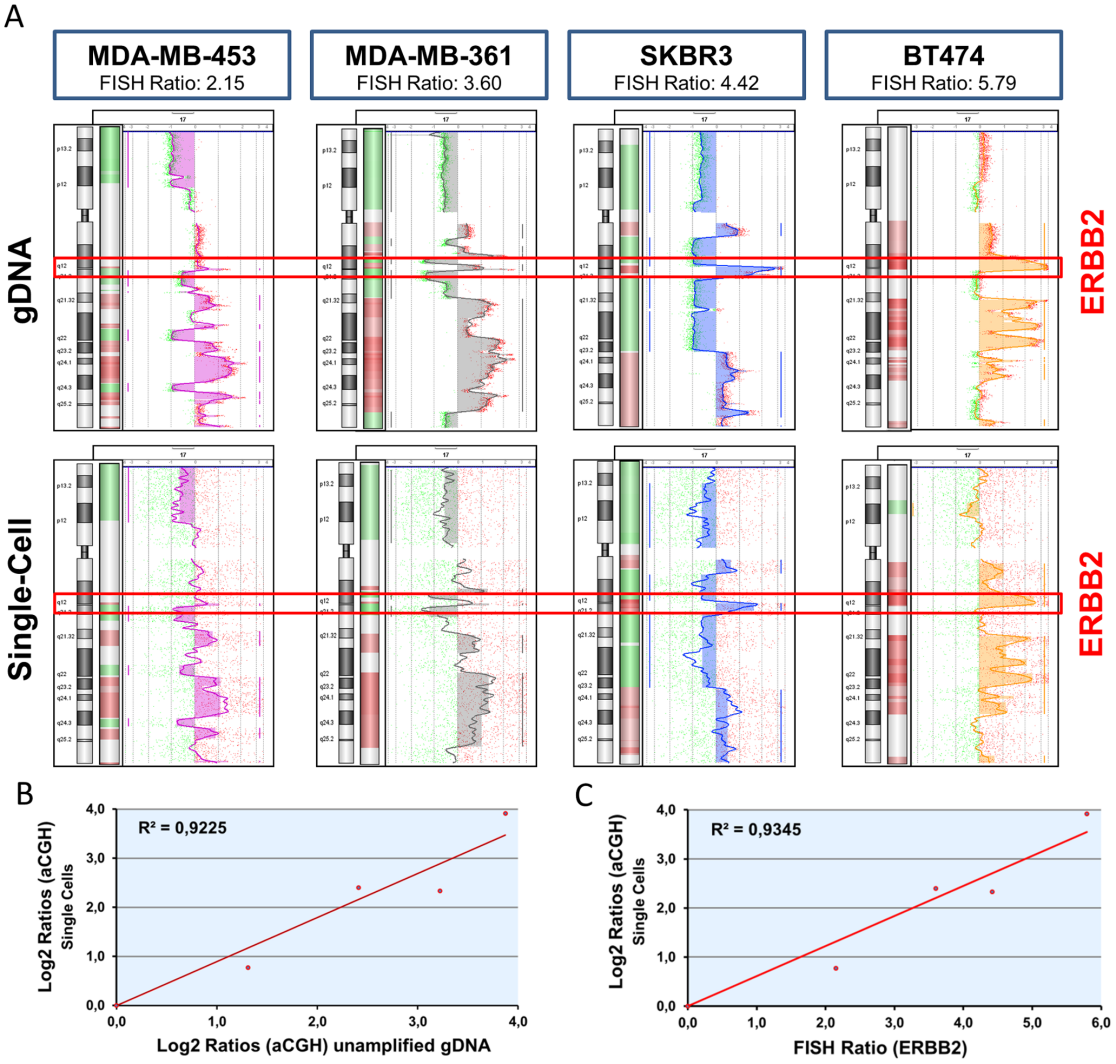
Quantitative assessment of copy number changes in tumor cells by single cell aCGH. A) Vertical aCGH profiles of chromosome 17 of four breast cancer cell lines with increasing copy number of ERBB2 locus (MDA-MB-453, MDA-MB-361, SKBR3 and BT474) generated using unamplified DNA (upper row) or single-cell WGA products (lower row). Red brackets indicate the position of the ERBB2 locus. Corresponding FISH ratios (ERBB2 vs. CEP17) of all cell lines are indicated in blue brackets. B) Correlation of average log2 values of ERBB2 specific probes obtained in single-cell aCGH experiments (Y-axis) vs. corresponding values obtained with unamplified DNA (X-axis). DNA samples from four breast cancer cell lines (MDA-MB-453, MDA-MB-361, SKBR3, BT474) have been included in the analysis. Pearson’s correlation coefficient 0.94. C) Correlation of average log2 values specific for ERBB2 locus obtained in single-cell aCGH experiments (Y-axis) vs. FISH ratios (ERBB2/CEP17) calculated for four breast cancer cell lines: MDA-MB-453, MDA-MB-361, SKBR3, BT474. Pearson’s correlation coefficient 0.97.

To further challenge our method, we asked whether we could correctly quantify copy number changes, i.e. assess the level of gene amplification in single cells. For this, we analyzed four different breast cancer cell lines (MDA-MB-453, MDA-MB-361, SK-BR-3 and BT-474) each representing different copy number of the ERBB2 locus (FISH ERBB2 to CEP17 ratios equal 2.15, 3.60, 4.42, 5.79, respectively). For each cell line both unamplified DNA and single-cell WGA products were analyzed by aCGH giving highly concordant profiles (Figure 4A). The average log2 ratios of probes representing the ERBB2 locus in single-cell aCGH experiments closely correlated with corresponding log2 values obtained for unamplified DNA and FISH ratios of ERBB2 (Pearson’s correlation coefficient 0.94 and 0.97, respectively, Figure 4 B-C).

### Application to single DCCs

**Figure 5:**
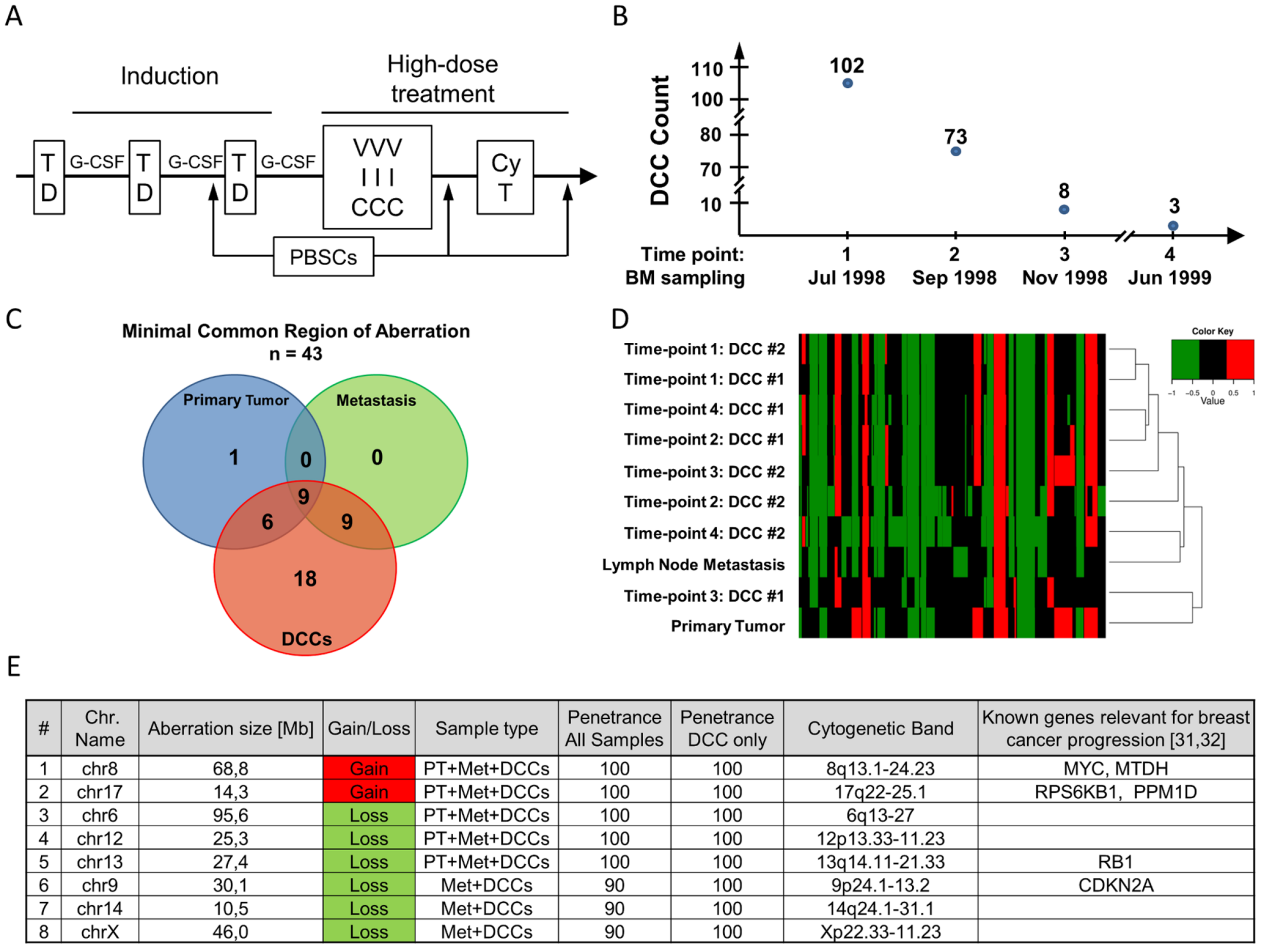
Molecular findings in individual DCCs over the course of systemic treatment. A) Chemotherapy regime: first patient was subjected to three cycles of 75 mg/m^2^ Taxotere® (T) and 50 mg/m^2^ Doxorubicin (D) in three week intervals. Subsequently followed two cycles of high dose chemotherapy treatment with an intermediate interval of 4-6 weeks. In the first cycle 500 mg/m^2^ of Vepesid (V), 4000 mg/m^2^ of Isofamid (I) and 500 mg/m^2^ of Carboplatin (C) was administered. The last cycle consisted of 1500 mg/m2 of Cyclophosphamid (Cy) and 200 mg/m2 of Thiotepa (T). Both cycles of high dose chemotherapeutic treatment were accompanied by addition of granulocyte colony-stimulating factor (G-CSF), and autologous transplant of peripheral blood stem cells (PBSCs). B) The course and outcome of bone marrow sampling. DCC count indicated the number of identified DCCs in 1.0×10^6^ mononuclear cells. C) Venn diagram depicting distribution of MRAs (gains and losses) across three types of clinical samples. D) Hierarchical clustering (distance: Euclidian; linkage: average) of samples including DCCs, the primary tumor and a metastatic lesion. E) Table depicting core MRAs that were found in all DCCs, the primary tumor and the metastatic lesion or in the metastatic compartments (DCCs and the lymph metastasis) only.

To test the assay on archival patient samples, we analyzed DCCs and corresponding metastatic tissue of an individual breast cancer patient. At the time of primary tumor resection (July 1998) this patient was diagnosed with stage IV breast cancer (PT4b, N1bii, pM1, G3, ER+, PR+, HER2-) with metastases detected in bones and lymph nodes and was subsequently treated with high dose chemotherapy (Figure 5A). During the course of treatment bone marrow was aspirated four times and screened for the presence of DCCs (Figure 5B). The DCC count was steadily decreasing throughout the treatment (Figure 5B), however it never reached zero. From each time point we analyzed two DCCs using our aCGH method. Additionally, DNA was collected from the primary tumor and a lymph node metastasis, amplified with *Ampli1™* WGA and subjected to aCGH. Subsequent aCGH analysis revealed multiple highly penetrant alterations among DCCs and corresponding tumor tissue samples (Figure S5). Systematic analysis of CNAs revealed 43 minimal regions of copy number alteration (MRAs), nine of which were shared by samples from the primary lesion, the lymph node metastasis and at least one of the DCCs (Figure 5C, Table S2 in the Text S1). Among the shared alterations, we identified five core MRAs shared by all samples (Figure 5E) – gains on 17q and 8q and losses on chromosomes 6, 12 and 13 – harboring multiple oncogenes and tumor suppressor genes, i.e. MYC, MTDH, RB1 and CDKN2A, that were previously associated with the progression of breast cancer [32], [33]. Additional three MRAs were shared between the metastatic tissue and all DCCs but not with the PT (Figure 5E). Hierarchical clustering revealed that 7 out of 8 DCCs were genetically different from the primary tumor and displayed more similarities to the lymph node metastasis (Figure 5D). The detailed inspection revealed substantial heterogeneity for various CNAs in the genomes of DCCs (Figure S5). One of the DCCs collected at the time point of the third bone marrow sampling (DCC #1, time point 3) lacked multiple aberrations shared by the other sister cells (Figure S5). This cell showed the highest genomic similarity to the primary tumor (Figures 5D), however it harbored fewer genomic alterations than the primary lesion (Table S3 in the Text S1). Hierarchical clustering confirmed the genomic divergence of cell DCC #1 from time point 3 in comparison with the remaining DCCs and the metastatic tissue. Lack of otherwise recurrent CNAs was also observed in DCC #2 from time point 4. In this case, however, the cell harbored additional unique copy number changes (both gains and losses), indicating the existence of subclones within the DCC population (Figure S5).

## Discussion

In this study, we established and validated a novel aCGH protocol for single cells enabling detection of chromosomal changes in single DCCs. This could be achieved by combined use of *Ampli1™* WGA technology together with customized DNA labeling technique and high-resolution Agilent SurePrint G3 Human 4×180 k oligonucleotide microarray platform. Data published by us and others indicate that *Ampli1™* technology is well suited for aCGH based analysis of DNA from single cells [25], [27] as well as DNA extracted form FFPE tissue [34]. Here, we show a new workflow for single-cell aCGH which, when applied on patient-derived clinical samples, appears to generate fewer artificial gains and losses than a recently published protocol [27].

First, we developed two novel PCR-based labeling approaches designed specifically to match the *Ampli1™* WGA products and compared it with recently published RP based labeling [27]. Direct comparison of the RP labeling with PCR-T2 technique provided highly comparable results in terms of sensitivity and reliability of both protocols. Importantly, the labeling techniques did not introduce any noticeable bias into the original WGA representation. In contrast to RP labeling, however, utilization of PCR-T2 technique requires only minute amount of DNA, leaving sufficient amount of the original WGA product for other high-throughput downstream application i.e. next generation sequencing (NGS). Moreover, the PCR-T2 procedure can be applied directly on the WGA products, while RP labeling requires a prior purification step. Hence, considering these practical advantages, we favor the PCR-T2 technique for processing *Ampli1™* WGA products.

Second, we sought to find the most suitable reference for single-cell aCGH analysis. Previous reports indicated that different biological nature of test and reference samples, i.e. amplified single-cell test and unamplified DNA, may influence both the sensitivity and the specificity of the single-cell aCGH assays [35]–[37]. These effects can be minimized by application of dedicated normalization algorithms [36], [38] or matched test and reference samples representing the same sample type – i.e. WGA products [27], [37]. Following this rationale, we determined that multiple pooled single-cell WGA products represent the optimal reference for single-cell aCGH experiments. This is in line with previous findings published by Bi and colleagues [26].

The third major improvement could be achieved by usage of high-resolution aCGH arrays based on oligonucleotides. Studies published in the recent years indicated applicability of the new generation of high-resolution SNP and CGH arrays for single-cell CGH analysis [26], [35], [39]–[43]. In comparison to the previously used BAC-based arrays [24], [25], [44] these technologies offer lower qualitative variability of the array slide manufacturing process, customizable microarray designs and very low probe spacing. Utilization of SurePrint G3 Agilent 4×180 k arrays (median probe spacing of 13 kb) enabled us to obtain high-quality aCGH results of single cells reaching precision comparable to the currently most sensitive single-cell aCGH protocol [27]. Despite that previous studies indicated that the detection limit of the single-cell aCGH may depend on the density of microarray probes [26], [39], the reproducible detection of alterations as small as 0.1 Mb suggests that 4×180 k arrays provide sufficient resolution.

Importantly, in our single-cell experiments we have not observed systemic fluctuations of log2 values of the microarray probes indicating a very homogeneous and unbiased representation of single-cell genomes. Therefore, in contrast to other single-cell aCGH approaches our method does not require custom design of copy number detection algorithms [36], [39] nor multiple hybridization runs to reliably detect copy number changes at the single-cell level [36]. Despite the increased levels of hybridization noise observed when processing single-cell DNA, standard aberrations recognition algorithms – ADM-2 and CBS [45] – provided high quality result. This is true even if old archival DNA (i.e. samples extracted from formalin fixed tissue) or re-amplified single-cell WGA products were used as a template for the analysis, showing high feasibility of the assay.

The resulting workflow proved to be remarkably sensitive allowing quantitative assessment of CNAs in single cells as demonstrated by the high correlation of signal intensity (represented by log2 ratios of the inspected locus) to copy numbers (measured as FISH value) when using single cell DNA. Moreover, our assay shows the ability to uncover cellular heterogeneity.

To date little is known about the level of heterogeneity of DCCs and their relation to the primary tumor and the metastatic lesion. Analysis of DCCs performed either directly or upon short-term culture provided already evidence for genomic variability of this cell population [12], [46], [47]. This may have clinical implications as variant cells may be subjected to natural selection [8]. Our results indicate that specific clones within the DCC population may be selected by high-dose chemotherapy. These clones (here represented by DCC #1 from time point 3 and DCC #2 from time point 4) are characterized by the presence of highly penetrant MRAs shared by PT, DCCs and metastasis, which either indicate close affinity to the founder cell of the tumor or suggest that such aberrations are essential for tumor formation at autochthonous and distant sites. This pattern of clonal selection was previously detected during the progression from myelodysplastic syndromes to secondary AML [48], [49]. In this model a minor subclone, harboring essential alterations, survives the selective sweep, gains additional mutations which enable its outgrowth leading to the clinical relapse [48], [49]. Our data indicate that this process may also be operative in breast cancer DCCs with gains on 17q and 8q, as well as losses on chromosomes 6, 12 and 13 representing stable genomic alterations that may constitute a *sine qua non* of tumor mass formation. Whether additional aberrations of key clones selected after chemotherapeutic treatment (DCC #1 from time point 3 and DCC #2 from time point 4) contribute to chemotherapy resistance awaits further studies. In summary, our analysis shows that DCCs in metastatic patients acquire heterogeneous aberrations on the basis of a stabilized aberrant genome with core changes. Such cells form dynamic cell populations from which different clones may be selected throughout the course of disease.

In conclusion, our new single-cell aCGH protocol enables reliable detection of CNAs in single cells. Further improvement of the achieved resolution may be possible by NGS technologies. Assisted by NGS-based targeted mutation analysis our assay will provide a precise but still affordable tool for monitoring the molecular evolution of early and advanced systemic cancer.

## Supporting Information

Figure S1 **Selection of best performing PCR-based DNA labeling technique.** A) Horizontal aCGH profiles of unamplified gDNA from PT-1590 cell (upper panel) and corresponding single-cell WGA product processed with PCR-T1 (middle panel) or PCR-T2 (lower panel). Red arrows indicate genomic intervals, which were called as aberrant in unamplified bulk gDNA but not in the single-cell WGA products. Green arrows indicate aberrant intervals detected exclusively in the single cell. B) Vertical aCGH profiles specific for chromosome 1 (placed in red brackets in the panel A) generated with unamplified gDNA (left panel) and single-cell WGA product processed with either PCR-T1 (middle panel) PCR-T2 (right panel). Arrows indicate genomic intervals that were falsely recognized as balanced when PCR-T1 labeling technique was used. C) Vertical aCGH profiles specific for chromosome 8 (placed in green brackets in the panel A) generated with unamplified gDNA (left panel) and single-cell WGA product processed with either PCR-T1 (middle panel) or PCR-T2 (right panel). Arrows indicate genomic intervals that were falsely recognized as balanced when PCR-T1 labeling technique was utilized. D) ROC-curves for single-cell aCGH comparing PCR-T1 vs. PCR-T2. Array CGH profile generated using unamplified gDNA of PT-1590 cells was taken as reference for the comparison. ROC analysis was performed on a genome-wide basis. A larger area under the curve (AUC) indicates higher accuracy of the method.
(TIF)

Figure S2 **Reproducibility of the single-cell aCGH assay.**A) Use of DNA from a cell pool as reference. B) Use of DNA from pooled single cells as reference. In both experiments horizontal aCGH profiles and corresponding correlation for the assessment of CNAs are shown. Experiments were performed on the same single-cell WGA product - PT-1590 single cell 3. Pearson’s correlation coefficient (r_xy_) was used to assess the reproducibility of the technical replicates.
(TIF)

Figure S3 **Array CGH using re-amplified single-cell WGA products.** A) Horizontal genome wide aCGH profiles of OE-19 cells generated using unamplified gDNA (upper panel) and a re-amplified single-cell WGA product labeled with PCR-T2 technique (middle panel) or RP labeling approach (lower panel). B) ROC-curves depicting the accuracy of the single-cell aCGH protocol when performed on re-amplified single-cell *Ampli1™* WGA products generated using OE-19 cells. Genomic profiles of unamplified gDNA of OE-19 cells was used for the comparison. ROC analysis was performed on a genome-wide basis. A larger area under the curve (AUC) indicates higher accuracy of the method. C) Horizontal genome wide aCGH profile of a single cell of a female donor hybridized against a male reference DNA (sex mismatch experiment).
(TIF)

Figure S4 **aCGH analysis of PT1590 esophageal cancer cell line cells.** Horizontal profiles of unamplified gDNA and single-cell WGA of PT1590 cells (depicted also in the Figure 3). Note the differences between the profiles of individual cells.
(TIF)

Figure S5 **aCGH profiles of clinical samples of a metastatic breast cancer patient.** Horizontal aCGH plots indicating the genomic gains and losses detected in eight DCCs and corresponding tumor tissue samples (primary tumor and lymph node metastases) of a patient with advanced breast cancer. Red arrows indicate genomic loci that remained balance in two selected cells, despite positively selected in the remaining samples. Blue arrows indicated genomic alterations occurring exclusively in DCC #2 from the time point 2.
(TIF)

Figure S6 **Comparison of PCR-T2 and RP-labeling approaches on immunostained cells from healthy donors.** Horizontal aCGH profiles of immunostained single-cell WGA products generated from white blood cells (WBCs) of three healthy individuals. Panels A, C, E depict single-cell samples processed with the RP-labeling technique, whereas panels B, D, F depict the same WGA samples processed with the PCR-T2 method. Aberrations were called using two aberration filters: (i) minimum number of probes in the region  =  10 and minimum absolute average log2 ration for a region  =  0.3 (upper panels); (ii) minimum number of probes in the region  =  25 and minimum absolute average log2 ration for a region  =  0.3 (lower panels). Black asterisks indicate genomic loci of randomly distributed false positive aberration calls. Green asterisks show locations of artifacts detected exclusively upon utilization of the RP-labeling technique.
(TIF)

Figure S7 **Comparison of PCR-T2 and RP-labeling approaches on clinical DCCs.** A, C) Horizontal aCGH profiles of immunostained single-cell WGA products generated using DCC of two prostate cancer patients. All single-cell were processed with the RP-labeling technique. B, D) Horizontal aCGH profiles of immunostained single-cell WGA products generated using DCC of two prostate cancer patients. All single-cell were processed with the PCR-T2 approach. Calling of genomic aberrations in A-D was performed using two aberration filters: (i) minimum number of probes in the region  =  10 and minimum absolute average log2 ration for a region  =  0.3 (upper panels); (ii) minimum number of probes in the region  =  25 and minimum absolute average log2 ration for a region  =  0.3 (lower panels). Black asterisks indicated genomic loci of randomly distributed false positive aberration calls. Green asterisks show locations of artifacts detected exclusively upon utilization of the RP-labeling technique.
(TIF)

Figure S8 **Direct comparison of single-cell cCGH with high-resolution aCGH.** A) Vertical profiles of single prostate cancer DCC generated with either cCGH (left panel) or high-resolution aCGH (right panel). Note that both profiles are very similar. B) Vertical aCGH profile specific for chromosome 6 of a prostate cancer DCC presented in the panel A. Red arrow indicates a DNA loss detected by both cCGH and aCGH, whereas black arrows show genomic loci at which alterations could by detected only with high-resolution aCGH. **Text S1. Tables S1-S3.** Table S1 in the Text S1. Comparison of hybridization characteristics resulting from the application of two PCR-based DNA labeling techniques (PCR-T1 and PCR-T2). Table S2 in the Text S1. Minimal Regions of Recurrent Copy Number Changes. Table S3 in the Text S1. Amount of aberrant intervals detected across the samples included in the case report study of an advanced breast cancer patient.
(TIF)

Text S1 **Tables S1-S3.** Table S1 in the Text S1. Comparison of hybridization characteristics resulting from the application of two PCR-based DNA labeling techniques (PCR-T1 and PCR-T2). Table S2 in the Text S1. Minimal Regions of Recurrent Copy Number Changes. Table S3 in the Text S1. Amount of aberrant intervals detected across the samples included in the case report study of an advanced breast cancer patient.
(DOCX)

## References

[1] Riethdorf S, Wikman H, Pantel K (2008) Review: Biological relevance of disseminated tumor cells in cancer patients. Int J Cancer 123: 1991–2006. 18712708 10.1002/ijc.23825

[2] Klein CA (2003) The systemic progression of human cancer: a focus on the individual disseminated cancer cell—the unit of selection. Adv Cancer Res 89: 35–67. 14587870 10.1016/s0065-230x(03)01002-9

[3] Cristofanilli M, Hayes DF, Budd GT, Ellis MJ, Stopeck A, et al (2005) Circulating tumor cells: a novel prognostic factor for newly diagnosed metastatic breast cancer. J Clin Oncol 23: 1420–1430. 15735118 10.1200/JCO.2005.08.140

[4] Cohen SJ, Punt CJ, Iannotti N, Saidman BH, Sabbath KD, et al (2008) Relationship of circulating tumor cells to tumor response, progression-free survival, and overall survival in patients with metastatic colorectal cancer. J Clin Oncol 26: 3213–3221. 18591556 10.1200/JCO.2007.15.8923

[5] Zhang L, Riethdorf S, Wu G, Wang T, Yang K, et al (2012) Meta-analysis of the prognostic value of circulating tumor cells in breast cancer. Clin Cancer Res 18: 5701–5710. 22908097 10.1158/1078-0432.CCR-12-1587

[6] Husemann Y, Geigl JB, Schubert F, Musiani P, Meyer M, et al (2008) Systemic spread is an early step in breast cancer. Cancer Cell 13: 58–68. 18167340 10.1016/j.ccr.2007.12.003

[7] Klein CA (2009) Parallel progression of primary tumours and metastases. Nat Rev Cancer 9: 302–312. 19308069 10.1038/nrc2627

[8] Klein CA (2013) Selection and adaptation during metastatic cancer progression. Nature 501: 365–372. 24048069 10.1038/nature12628

[9] Schardt JA, Meyer M, Hartmann CH, Schubert F, Schmidt-Kittler O, et al (2005) Genomic analysis of single cytokeratin-positive cells from bone marrow reveals early mutational events in breast cancer. Cancer Cell 8: 227–239. 16169467 10.1016/j.ccr.2005.08.003

[10] Schmidt-Kittler O, Ragg T, Daskalakis A, Granzow M, Ahr A, et al (2003) From latent disseminated cells to overt metastasis: genetic analysis of systemic breast cancer progression. Proc Natl Acad Sci U S A 100: 7737–7742. 12808139 10.1073/pnas.1331931100 PMC164657

[11] Stoecklein NH, Hosch SB, Bezler M, Stern F, Hartmann CH, et al (2008) Direct genetic analysis of single disseminated cancer cells for prediction of outcome and therapy selection in esophageal cancer. Cancer Cell 13: 441–453. 18455127 10.1016/j.ccr.2008.04.005

[12] Klein CA, Blankenstein TJ, Schmidt-Kittler O, Petronio M, Polzer B, et al (2002) Genetic heterogeneity of single disseminated tumour cells in minimal residual cancer. Lancet 360: 683–689. 12241875 10.1016/S0140-6736(02)09838-0

[13] Janni W, Vogl FD, Wiedswang G, Synnestvedt M, Fehm T, et al (2011) Persistence of disseminated tumor cells in the bone marrow of breast cancer patients predicts increased risk for relapse--a European pooled analysis. Clin Cancer Res 17: 2967–2976. 21415211 10.1158/1078-0432.CCR-10-2515

[14] Pierga JY, Hajage D, Bachelot T, Delaloge S, Brain E, et al (2012) High independent prognostic and predictive value of circulating tumor cells compared with serum tumor markers in a large prospective trial in first-line chemotherapy for metastatic breast cancer patients. Ann Oncol 23: 618–624. 21642515 10.1093/annonc/mdr263

[15] Zhang L, Cui X, Schmitt K, Hubert R, Navidi W, et al (1992) Whole genome amplification from a single cell: implications for genetic analysis. Proc Natl Acad Sci U S A 89: 5847–5851. 1631067 10.1073/pnas.89.13.5847 PMC49394

[16] Lage JM, Leamon JH, Pejovic T, Hamann S, Lacey M, et al (2003) Whole genome analysis of genetic alterations in small DNA samples using hyperbranched strand displacement amplification and array-CGH. Genome Res 13: 294–307. 12566408 10.1101/gr.377203 PMC420367

[17] Spits C, Le Caignec C, De Rycke M, Van Haute L, Van Steirteghem A, et al (2006) Whole-genome multiple displacement amplification from single cells. Nat Protoc 1: 1965–1970. 17487184 10.1038/nprot.2006.326

[18] Munne S, Fragouli E, Colls P, Katz-Jaffe M (2010) Schoolcraft W, et al (2010) Improved detection of aneuploid blastocysts using a new 12-chromosome FISH test. Reprod Biomed Online 20: 92–97. 20158993 10.1016/j.rbmo.2009.10.015

[19] Klein CA, Schmidt-Kittler O, Schardt JA, Pantel K, Speicher MR, et al (1999) Comparative genomic hybridization, loss of heterozygosity, and DNA sequence analysis of single cells. Proc Natl Acad Sci U S A 96: 4494–4499. 10200290 10.1073/pnas.96.8.4494 PMC16360

[20] Zhong XB, Lizardi PM, Huang XH, Bray-Ward PL, Ward DC (2001) Visualization of oligonucleotide probes and point mutations in interphase nuclei and DNA fibers using rolling circle DNA amplification. Proc Natl Acad Sci U S A 98: 3940–3945. 11274414 10.1073/pnas.061026198 PMC31158

[21] Traversa MV, Carey L, Leigh D (2010) A molecular strategy for routine preimplantation genetic diagnosis in both reciprocal and Robertsonian translocation carriers. Mol Hum Reprod 16: 329–337. 20172975 10.1093/molehr/gaq013

[22] Lu S, Zong C, Fan W, Yang M, Li J, et al (2012) Probing meiotic recombination and aneuploidy of single sperm cells by whole-genome sequencing. Science 338: 1627–1630. 23258895 10.1126/science.1229112 PMC3590491

[23] Zong C, Lu S, Chapman AR, Xie XS (2012) Genome-wide detection of single-nucleotide and copy-number variations of a single human cell. Science 338: 1622–1626. 23258894 10.1126/science.1229164 PMC3600412

[24] Fiegler H, Geigl JB, Langer S, Rigler D, Porter K, et al (2007) High resolution array-CGH analysis of single cells. Nucleic Acids Res 35: e15. 17178751 10.1093/nar/gkl1030 PMC1807964

[25] Fuhrmann C, Schmidt-Kittler O, Stoecklein NH, Petat-Dutter K, Vay C, et al (2008) High-resolution array comparative genomic hybridization of single micrometastatic tumor cells. Nucleic Acids Res 36: e39. 18344524 10.1093/nar/gkn101 PMC2367728

[26] Bi W, Breman A, Shaw CA, Stankiewicz P, Gambin T, et al (2012) Detection of >/ = 1 Mb microdeletions and microduplications in a single cell using custom oligonucleotide arrays. Prenat Diagn 32: 10–20. 22470934 10.1002/pd.2855

[27] Mohlendick B, Bartenhagen C, Behrens B, Honisch E, Raba K, et al (2013) A Robust Method to Analyze Copy Number Alterations of Less than 100 kb in Single Cells Using Oligonucleotide Array CGH. PLoS One 8: e67031. 23825608 10.1371/journal.pone.0067031 PMC3692546

[28] Hosch S, Kraus J, Scheunemann P, Izbicki JR, Schneider C, et al (2000) Malignant potential and cytogenetic characteristics of occult disseminated tumor cells in esophageal cancer. Cancer Res 60: 6836–6840. 11156375

[29] Braun S, Pantel K, Muller P, Janni W, Hepp F, et al (2000) Cytokeratin-positive cells in the bone marrow and survival of patients with stage I, II, or III breast cancer. N Engl J Med 342: 525–533. 10684910 10.1056/NEJM200002243420801

[30] Fehm T, Braun S, Muller V, Janni W, Gebauer G, et al (2006) A concept for the standardized detection of disseminated tumor cells in bone marrow from patients with primary breast cancer and its clinical implementation. Cancer 107: 885–892. 16874814 10.1002/cncr.22076

[31] Mathiesen RR, Fjelldal R, Liestol K, Due EU, Geigl JB, et al (2012) High-resolution analyses of copy number changes in disseminated tumor cells of patients with breast cancer. Int J Cancer 131: E405–415. 21935921 10.1002/ijc.26444

[32] Futreal PA, Coin L, Marshall M, Down T, Hubbard T, et al (2004) A census of human cancer genes. Nat Rev Cancer 4: 177–183. 14993899 10.1038/nrc1299 PMC2665285

[33] Santarius T, Shipley J, Brewer D, Stratton MR, Cooper CS (2010) A census of amplified and overexpressed human cancer genes. Nat Rev Cancer 10: 59–64. 20029424 10.1038/nrc2771

[34] Arneson N, Moreno J, Iakovlev V, Ghazani A, Warren K, et al (2012) Comparison of whole genome amplification methods for analysis of DNA extracted from microdissected early breast lesions in formalin-fixed paraffin-embedded tissue. ISRN Oncol 2012: 710692. 22530150 10.5402/2012/710692 PMC3317021

[35] Iwamoto K, Bundo M, Ueda J, Nakano Y, Ukai W, et al (2007) Detection of chromosomal structural alterations in single cells by SNP arrays: a systematic survey of amplification bias and optimized workflow. PLoS One 2: e1306. 18074030 10.1371/journal.pone.0001306 PMC2111048

[36] Konings P, Vanneste E, Jackmaert S, Ampe M, Verbeke G, et al (2012) Microarray analysis of copy number variation in single cells. Nat Protoc 7: 281–310. 22262009 10.1038/nprot.2011.426

[37] Ling J, Zhuang G, Tazon-Vega B, Zhang C, Cao B, et al (2009) Evaluation of genome coverage and fidelity of multiple displacement amplification from single cells by SNP array. Mol Hum Reprod 15: 739–747. 19671595 10.1093/molehr/gap066 PMC2762374

[38] Cheng J, Vanneste E, Konings P, Voet T, Vermeesch JR, et al (2011) Single-cell copy number variation detection. Genome Biol 12: R80. 21854607 10.1186/gb-2011-12-8-r80 PMC3245619

[39] Geigl JB, Obenauf AC, Waldispuehl-Geigl J, Hoffmann EM, Auer M, et al (2009) Identification of small gains and losses in single cells after whole genome amplification on tiling oligo arrays. Nucleic Acids Res 37: e105. 19541849 10.1093/nar/gkp526 PMC2731907

[40] Hellani A, Abu-Amero K, Azouri J, El-Akoum S (2008) Successful pregnancies after application of array-comparative genomic hybridization in PGS-aneuploidy screening. Reprod Biomed Online 17: 841–847. 19079969 10.1016/s1472-6483(10)60413-0

[41] Johnson DS, Gemelos G, Baner J, Ryan A, Cinnioglu C, et al (2010) Preclinical validation of a microarray method for full molecular karyotyping of blastomeres in a 24-h protocol. Hum Reprod 25: 1066–1075. 20100701 10.1093/humrep/dep452 PMC2839907

[42] Treff NR, Su J, Tao X, Levy B, Scott RT Jr (2010) Accurate single cell 24 chromosome aneuploidy screening using whole genome amplification and single nucleotide polymorphism microarrays. Fertil Steril 94: 2017–2021. 20188357 10.1016/j.fertnstert.2010.01.052

[43] Vanneste E, Voet T, Le Caignec C, Ampe M, Konings P, et al (2009) Chromosome instability is common in human cleavage-stage embryos. Nat Med 15: 577–583. 19396175 10.1038/nm.1924

[44] Le Caignec C, Spits C, Sermon K, De Rycke M, Thienpont B, et al (2006) Single-cell chromosomal imbalances detection by array CGH. Nucleic Acids Res 34: e68. 16698960 10.1093/nar/gkl336 PMC3303179

[45] Olshen AB, Venkatraman ES, Lucito R, Wigler M (2004) Circular binary segmentation for the analysis of array-based DNA copy number data. Biostatistics 5: 557–572. 15475419 10.1093/biostatistics/kxh008

[46] Gangnus R, Langer S, Breit E, Pantel K, Speicher MR (2004) Genomic profiling of viable and proliferative micrometastatic cells from early-stage breast cancer patients. Clin Cancer Res 10: 3457–3464. 15161702 10.1158/1078-0432.CCR-03-0818

[47] Kraus J, Pantel K, Pinkel D, Albertson DG, Speicher MR (2003) High-resolution genomic profiling of occult micrometastatic tumor cells. Genes Chromosomes Cancer 36: 159–166. 12508244 10.1002/gcc.10160

[48] Ding L, Ley TJ, Larson DE, Miller CA, Koboldt DC, et al (2012) Clonal evolution in relapsed acute myeloid leukaemia revealed by whole-genome sequencing. Nature 481: 506–510. 22237025 10.1038/nature10738 PMC3267864

[49] Parkin B, Ouillette P, Li Y, Keller J, Lam C, et al (2013) Clonal evolution and devolution after chemotherapy in adult acute myelogenous leukemia. Blood 121: 369–377. 23175688 10.1182/blood-2012-04-427039 PMC3653567

