# Supplementary figures and images for: Reliable Single Cell Array CGH for Clinical Samples
Source: PLoS One. 2014 Jan 21;9(1):e85907. doi: 10.1371/journal.pone.0085907 (PMC3897541; doi:10.1371/journal.pone.0085907)

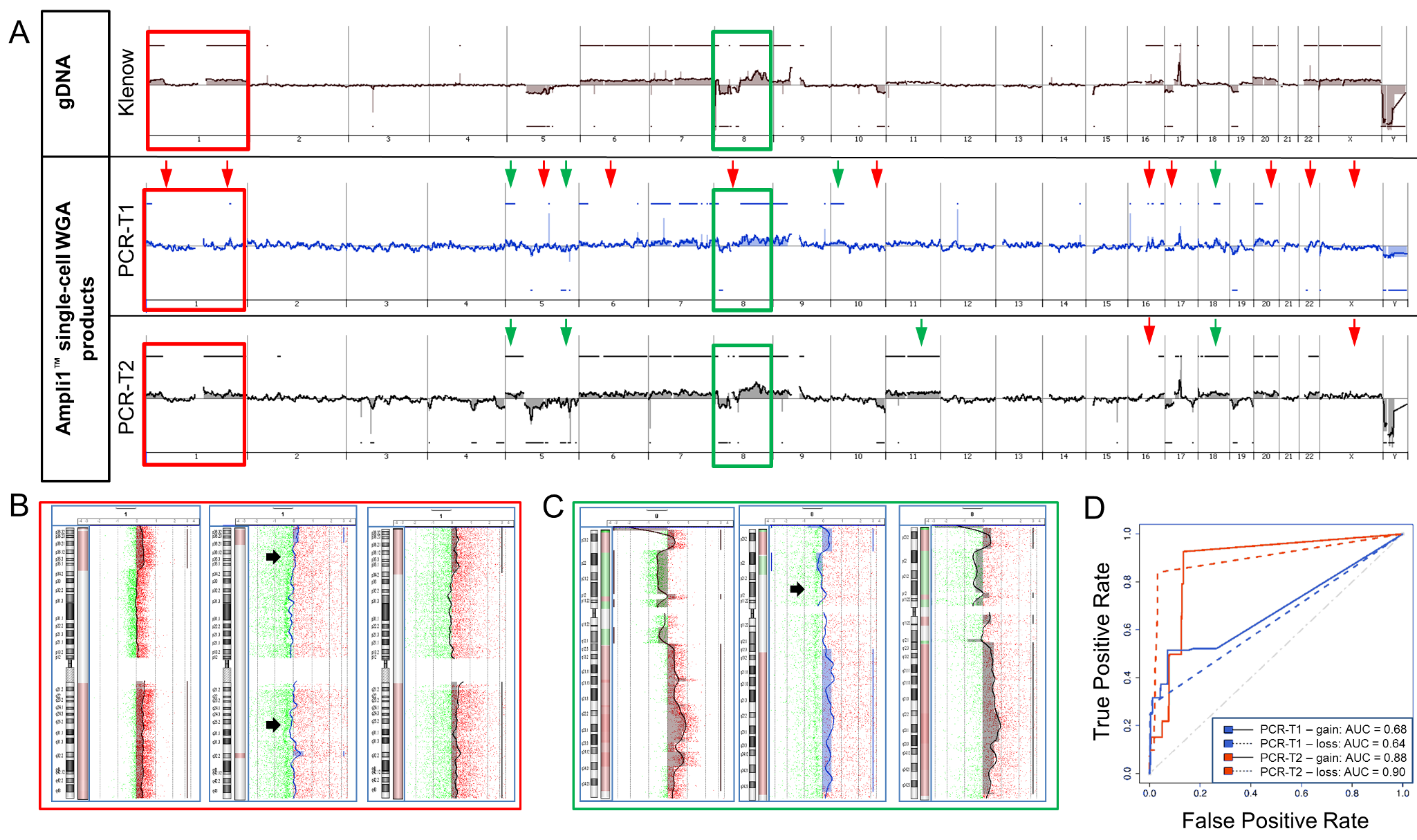

Supplement: Figure S1 — Selection of best performing PCR-based DNA labeling technique. A) Horizontal aCGH profiles of unamplified gDNA from PT-1590 cell (upper panel) and corresponding single-cell WGA product processed with PCR-T1 (middle panel) or PCR-T2 (lower panel). Red arrows indicate genomic intervals, which were called as aberrant in unamplified bulk gDNA but not in the single-cell WGA products. Green arrows indicate aberrant intervals detected exclusively in the single cell. B) Vertical aCGH profiles specific for chromosome 1 (placed in red brackets in the panel A) generated with unamplified gDNA (left panel) and single-cell WGA product processed with either PCR-T1 (middle panel) PCR-T2 (right panel). Arrows indicate genomic intervals that were falsely recognized as balanced when PCR-T1 labeling technique was used. C) Vertical aCGH profiles specific for chromosome 8 (placed in green brackets in the panel A) generated with unamplified gDNA (left panel) and single-cell WGA product processed with either PCR-T1 (middle panel) or PCR-T2 (right panel). Arrows indicate genomic intervals that were falsely recognized as balanced when PCR-T1 labeling technique was utilized. D) ROC-curves for single-cell aCGH comparing PCR-T1 vs. PCR-T2. Array CGH profile generated using unamplified gDNA of PT-1590 cells was taken as reference for the comparison. ROC analysis was performed on a genome-wide basis. A larger area under the curve (AUC) indicates higher accuracy of the method. (TIF) [file pone.0085907.s001.tif]

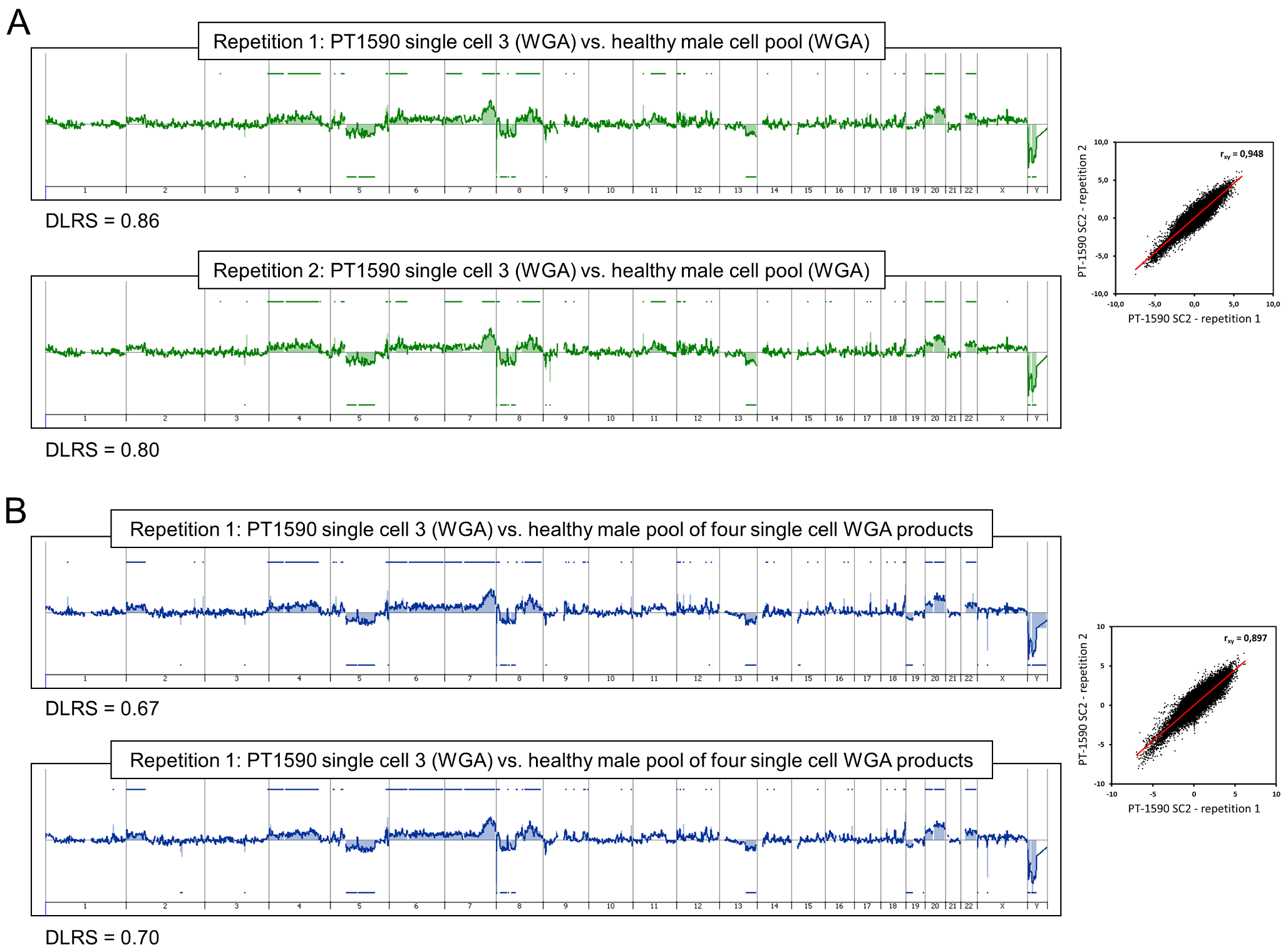

Supplement: Figure S2 — Reproducibility of the single-cell aCGH assay.A) Use of DNA from a cell pool as reference. B) Use of DNA from pooled single cells as reference. In both experiments horizontal aCGH profiles and corresponding correlation for the assessment of CNAs are shown. Experiments were performed on the same single-cell WGA product - PT-1590 single cell 3. Pearson’s correlation coefficient (rxy) was used to assess the reproducibility of the technical replicates. (TIF) [file pone.0085907.s002.tif]

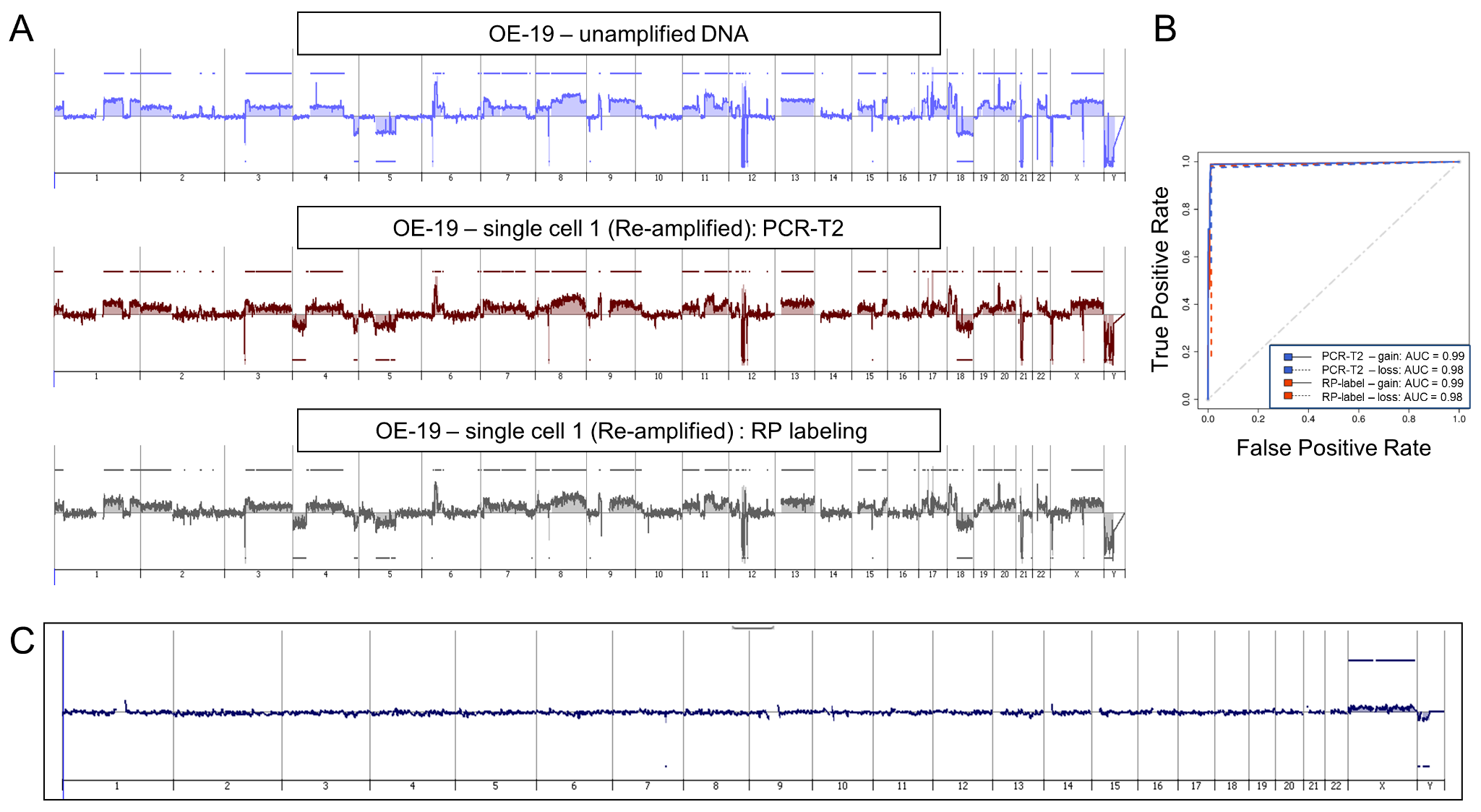

Supplement: Figure S3 — Array CGH using re-amplified single-cell WGA products. A) Horizontal genome wide aCGH profiles of OE-19 cells generated using unamplified gDNA (upper panel) and a re-amplified single-cell WGA product labeled with PCR-T2 technique (middle panel) or RP labeling approach (lower panel). B) ROC-curves depicting the accuracy of the single-cell aCGH protocol when performed on re-amplified single-cell Ampli1™ WGA products generated using OE-19 cells. Genomic profiles of unamplified gDNA of OE-19 cells was used for the comparison. ROC analysis was performed on a genome-wide basis. A larger area under the curve (AUC) indicates higher accuracy of the method. C) Horizontal genome wide aCGH profile of a single cell of a female donor hybridized against a male reference DNA (sex mismatch experiment). (TIF) [file pone.0085907.s003.tif]

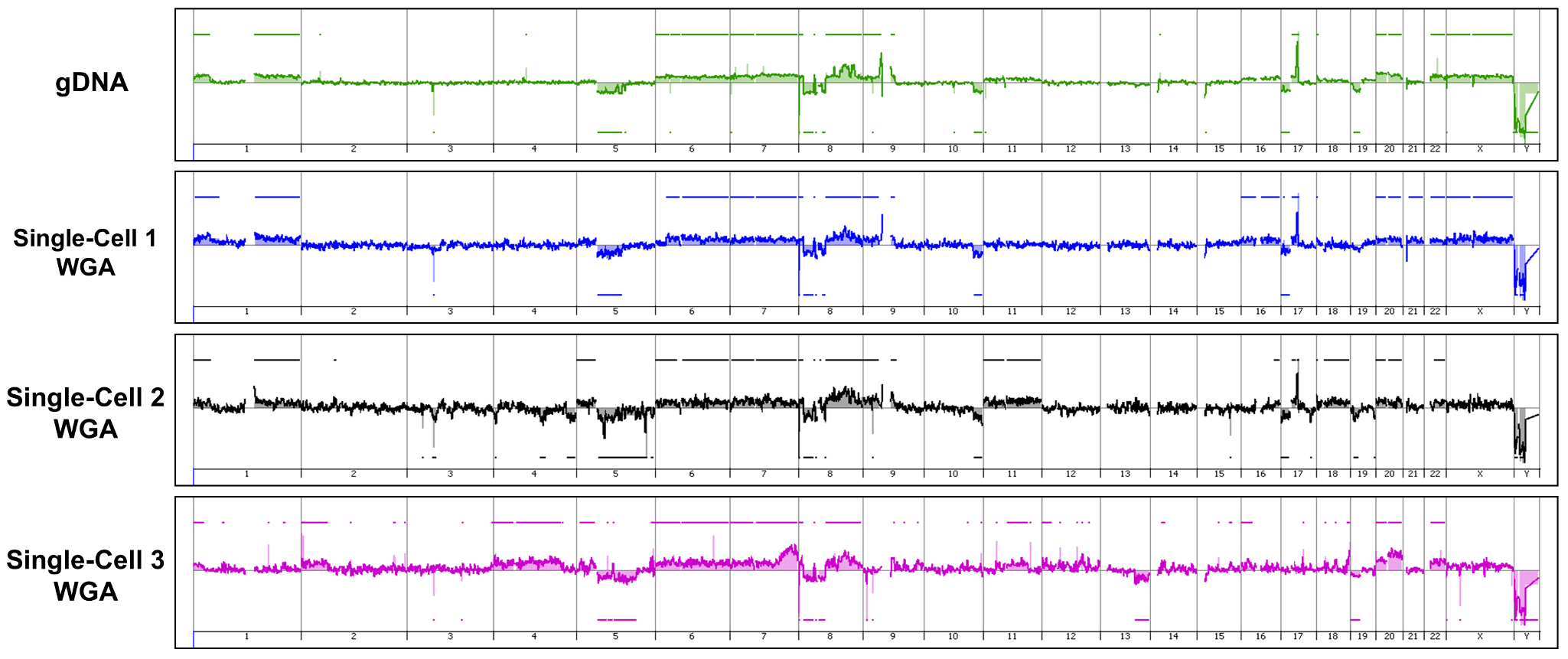

Supplement: Figure S4 — aCGH analysis of PT1590 esophageal cancer cell line cells. Horizontal profiles of unamplified gDNA and single-cell WGA of PT1590 cells (depicted also in the Figure 3). Note the differences between the profiles of individual cells. (TIF) [file pone.0085907.s004.tif]

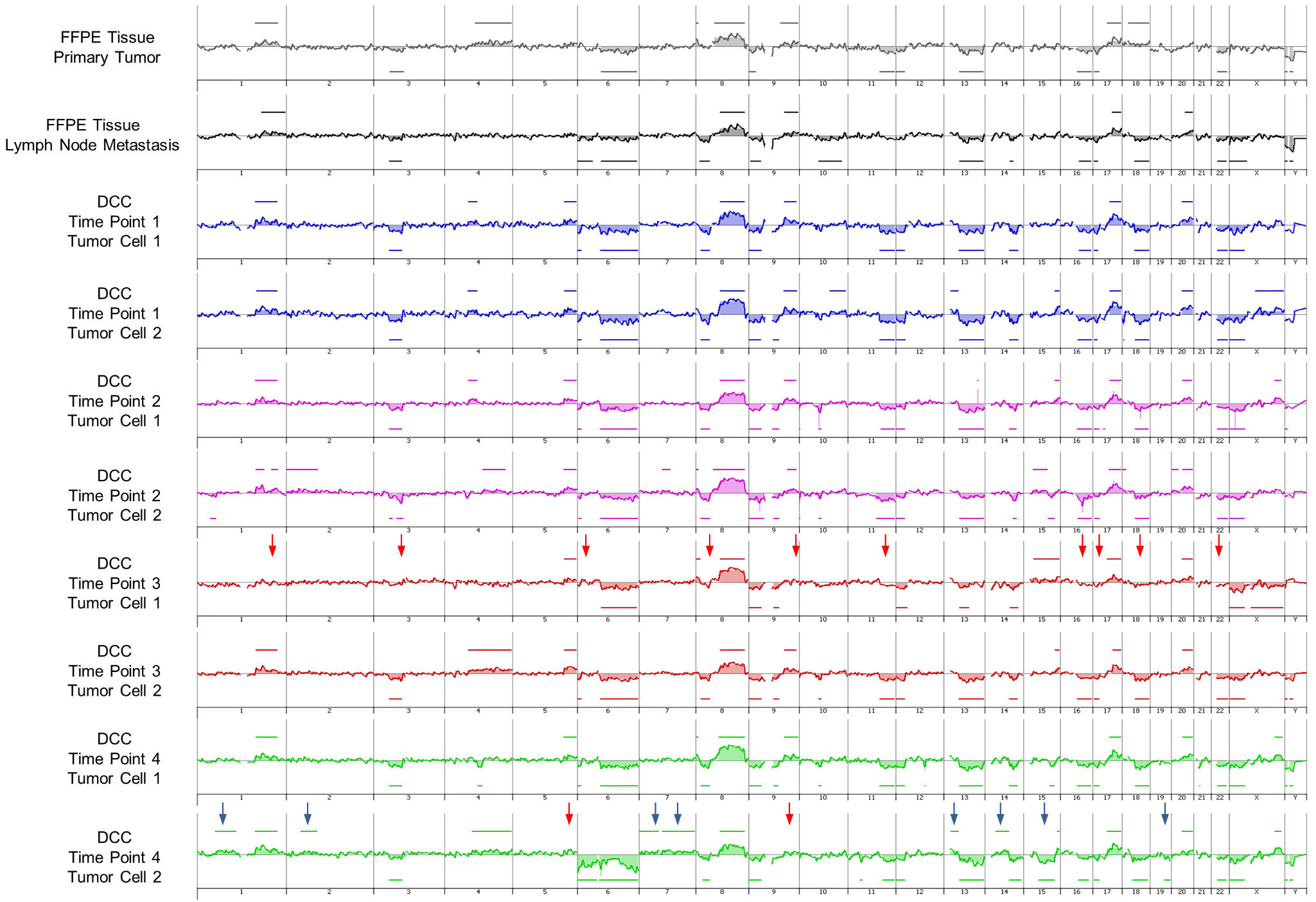

Supplement: Figure S5 — aCGH profiles of clinical samples of a metastatic breast cancer patient. Horizontal aCGH plots indicating the genomic gains and losses detected in eight DCCs and corresponding tumor tissue samples (primary tumor and lymph node metastases) of a patient with advanced breast cancer. Red arrows indicate genomic loci that remained balance in two selected cells, despite positively selected in the remaining samples. Blue arrows indicated genomic alterations occurring exclusively in DCC #2 from the time point 2. (TIF) [file pone.0085907.s005.tif]

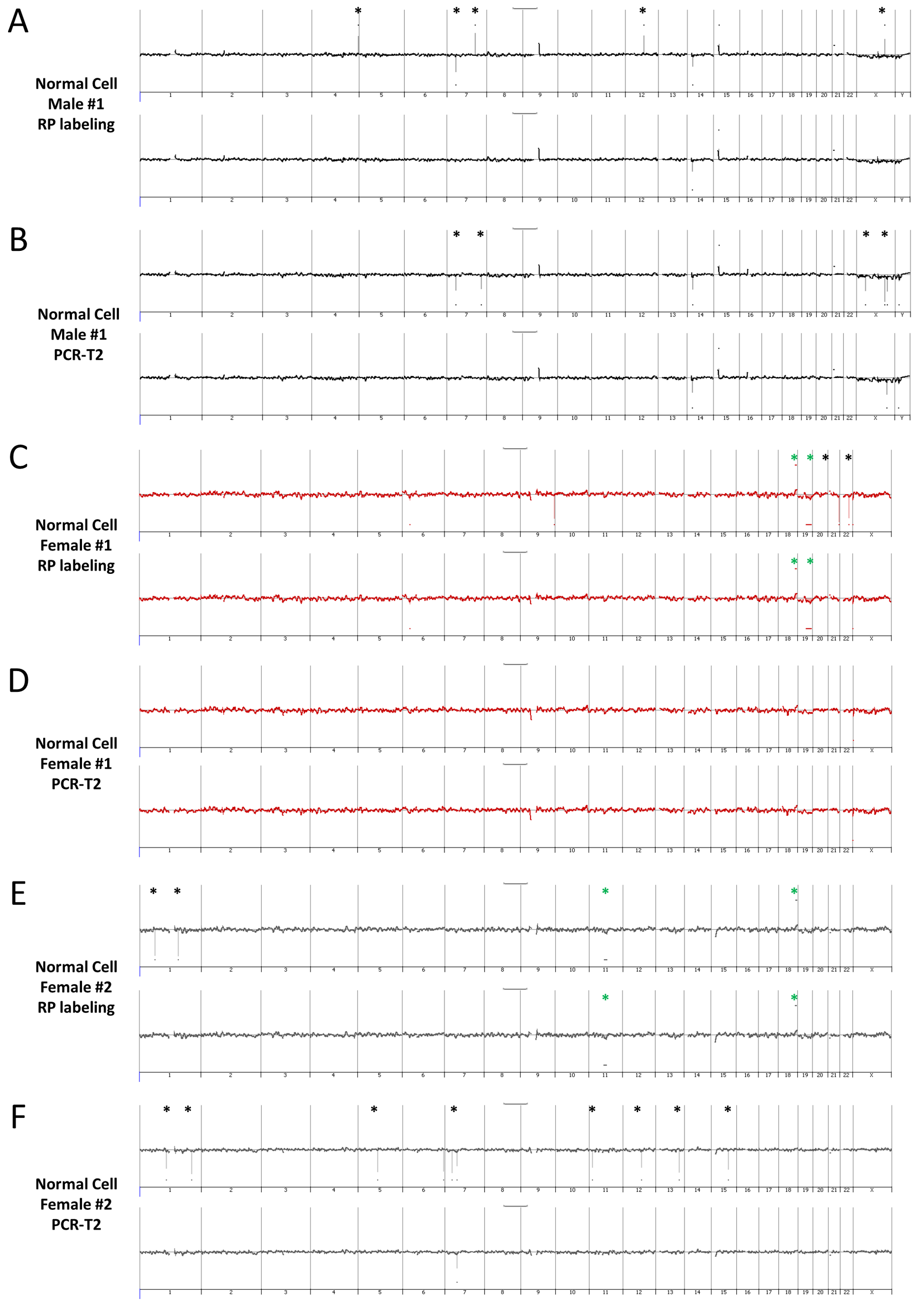

Supplement: Figure S6 — Comparison of PCR-T2 and RP-labeling approaches on immunostained cells from healthy donors. Horizontal aCGH profiles of immunostained single-cell WGA products generated from white blood cells (WBCs) of three healthy individuals. Panels A, C, E depict single-cell samples processed with the RP-labeling technique, whereas panels B, D, F depict the same WGA samples processed with the PCR-T2 method. Aberrations were called using two aberration filters: (i) minimum number of probes in the region = 10 and minimum absolute average log2 ration for a region = 0.3 (upper panels); (ii) minimum number of probes in the region = 25 and minimum absolute average log2 ration for a region = 0.3 (lower panels). Black asterisks indicate genomic loci of randomly distributed false positive aberration calls. Green asterisks show locations of artifacts detected exclusively upon utilization of the RP-labeling technique. (TIF) [file pone.0085907.s006.tif]

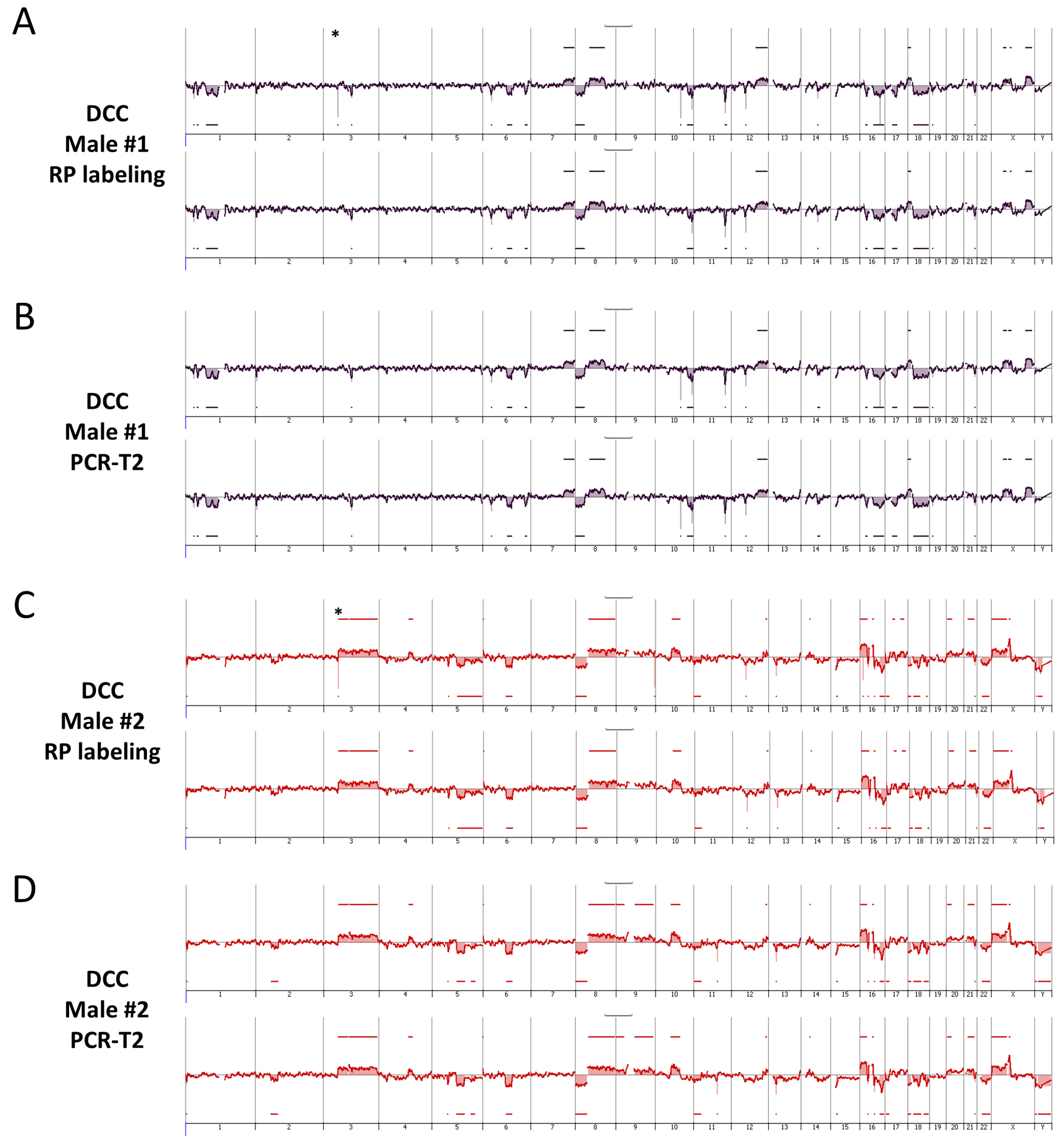

Supplement: Figure S7 — Comparison of PCR-T2 and RP-labeling approaches on clinical DCCs. A, C) Horizontal aCGH profiles of immunostained single-cell WGA products generated using DCC of two prostate cancer patients. All single-cell were processed with the RP-labeling technique. B, D) Horizontal aCGH profiles of immunostained single-cell WGA products generated using DCC of two prostate cancer patients. All single-cell were processed with the PCR-T2 approach. Calling of genomic aberrations in A-D was performed using two aberration filters: (i) minimum number of probes in the region = 10 and minimum absolute average log2 ration for a region = 0.3 (upper panels); (ii) minimum number of probes in the region = 25 and minimum absolute average log2 ration for a region = 0.3 (lower panels). Black asterisks indicated genomic loci of randomly distributed false positive aberration calls. Green asterisks show locations of artifacts detected exclusively upon utilization of the RP-labeling technique. (TIF) [file pone.0085907.s007.tif]

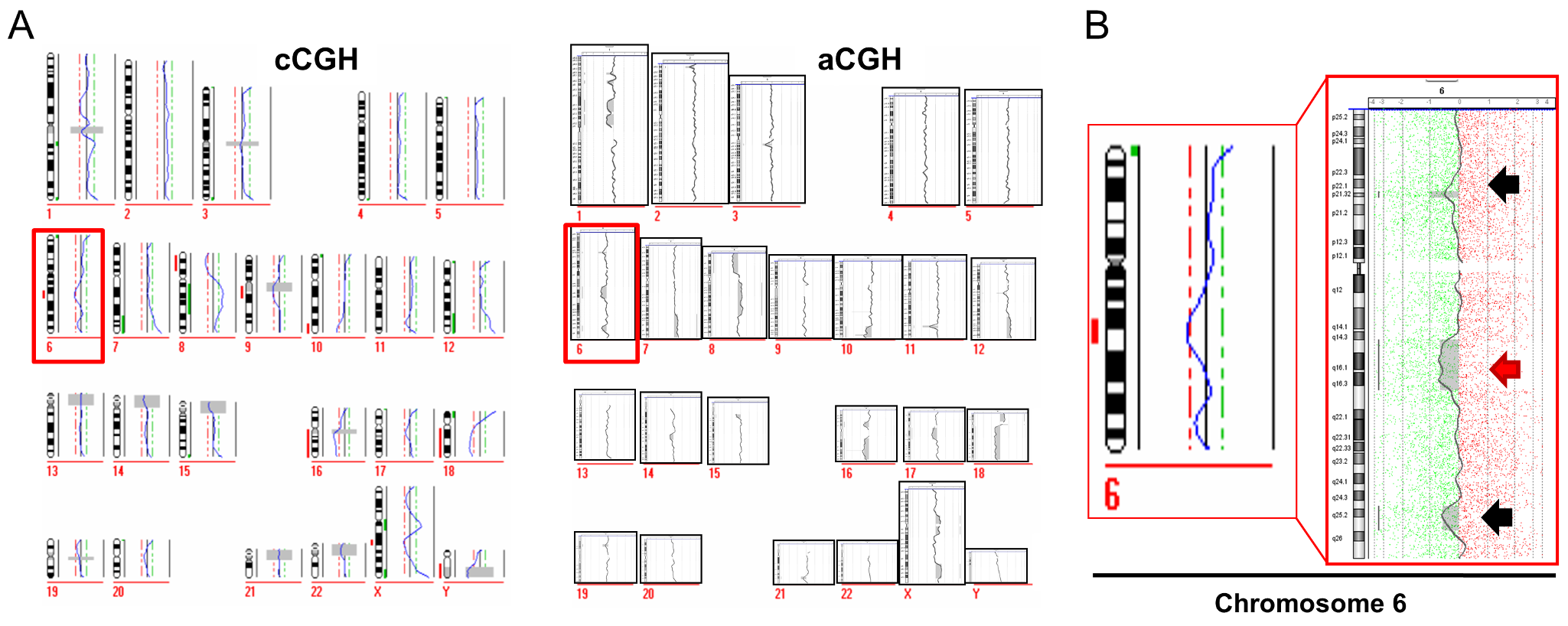

Supplement: Figure S8 — Direct comparison of single-cell cCGH with high-resolution aCGH. A) Vertical profiles of single prostate cancer DCC generated with either cCGH (left panel) or high-resolution aCGH (right panel). Note that both profiles are very similar. B) Vertical aCGH profile specific for chromosome 6 of a prostate cancer DCC presented in the panel A. Red arrow indicates a DNA loss detected by both cCGH and aCGH, whereas black arrows show genomic loci at which alterations could by detected only with high-resolution aCGH. Text S1. Tables S1-S3. Table S1 in the Text S1. Comparison of hybridization characteristics resulting from the application of two PCR-based DNA labeling techniques (PCR-T1 and PCR-T2). Table S2 in the Text S1. Minimal Regions of Recurrent Copy Number Changes. Table S3 in the Text S1. Amount of aberrant intervals detected across the samples included in the case report study of an advanced breast cancer patient. (TIF) [file pone.0085907.s008.tif]
