# Supplementary material for: Reliable Single Cell Array CGH for Clinical Samples
Source: PLoS One. 2014 Jan 21;9(1):e85907. doi: 10.1371/journal.pone.0085907 (PMC3897541; doi:10.1371/journal.pone.0085907)
Supplement: Text S1 — Tables S1-S3. Table S1 in the Text S1. Comparison of hybridization characteristics resulting from the application of two PCR-based DNA labeling techniques (PCR-T1 and PCR-T2). Table S2 in the Text S1. Minimal Regions of Recurrent Copy Number Changes. Table S3 in the Text S1. Amount of aberrant intervals detected across the samples included in the case report study of an advanced breast cancer patient. (DOCX) [file pone.0085907.s009.docx]

**Supplementary text**

***Performance on single cells isolated from clinical samples***

Fixation and staining methods used to detect disseminated cancer cells (DCCs) may damage the single-cell genomic DNA and jeopardize the success of subsequent downstream analysis. For this reason, we tested RP-labeling and PCR-T2 for their reliability in the analysis of pre-processed single cells. For this purpose the mononuclear cell fraction was isolated from peripheral blood of four healthy individuals and stained for vimentin utilizing the same immunocytochemistry staining protocol as used for detection of DCCs. Subsequently, single cells were isolated, subjected to WGA and analyzed by aCGH. Inspection of the resulting aCGH profiles revealed presence of focal changes of log2 values spread across the single-cell genomes manifested in the results as small gain and losses (Figure S6 and S7). Since these events occurred at random positions in the genome irrespective of the labeling technique they were classified as false positive aberration calls. To avoid their detection the settings of the aberration filter were modified so that the minimal aberration had to encompass a minimum of 25 consecutive probes along a chromosome (Figure S6 and S7). This approach, however, did not guarantee the removal of all false positive aberrations detected after samples that were processed with the RP-labeling approach (Figure S6). Taking this into consideration, PCR-T2 was used as our method of choice for subsequent experiments based on its practical characteristics.

To assess the reliability of the new aCGH assay in DCCs its outcome was validated with results obtained using chromosomal CGH (cCGH). A panel of four archival single DCCs obtained from late stage prostate cancer patients was processed with both aCGH and cCGH. We found highly concordant aberration profiles of the cCGH and aCGH experiments (Figure S8A). Nevertheless, aCGH proved to be much more sensitive and allowed detection of genomic aberrations that were beyond the scope of cCGH (Supplementary Figure S8B).

Table S1. Comparison of hybridization characteristics resulting from the application of two PCR-based DNA labeling techniques (PCR-T1 and PCR-T2).

|  | **PT-1590** | | | | **Healthy Donor** | | | |
| --- | --- | --- | --- | --- | --- | --- | --- | --- |
|  | Cell Pool | | Single Cell | | Cell Pool | | Single Cell | |
|  | PCR-T1 | PCR-T2 | PCR-T1 | PCR-T2 | PCR-T1 | PCR-T2 | PCR-T1 | PCR-T2 |
| DNA yield [µg] - Test | 4.0 | 15.0 | 5.6 | 18.3 | 4.8 | 14.4 | 4.1 | 18.8 |
| Dye Incorporation Test (Cy5) | 67.3 | 8.8 | 42.1 | 10.5 | 47.0 | 7.4 | 50.0 | 10.9 |
| DNA yield [µg] - Reference | 4.7 | 16.7 | 4.5 | 18.1 | 4.7 | 17.9 | 4.3 | 18.9 |
| Dye Incorporation Reference (Cy3) | 67.0 | 5.5 | 56.0 | 5.5 | 55.0 | 6.3 | 62.0 | 6.2 |
| Signal Intensity - Red | 214 | 1169 | 273 | 1751 | 174 | 891 | 106 | 979 |
| Signal Intensity - Green | 3.0 | 48.8 | 4.5 | 63.7 | 1.6 | 22.7 | 1.3 | 3.7 |
| Signal-to Noise Ratio - Red | 43 | 290 | 50 | 389 | 36 | 305 | 39 | 404 |
| Signal-to-Noise Ratio - Green | 1.9 | 32.2 | 2.7 | 56.9 | 1.5 | 26.7 | 1.5 | 9.4 |

Table S2. Minimal Regions of Recurrent Copy Number Changes.

| **#** | **Chr Name** | **Aberration size [Mb]** | **Gain/Loss** | **Sample type** | **Penetrance All Samples** | **Penetrance DCC only** | **Cytogenetic band** |
| --- | --- | --- | --- | --- | --- | --- | --- |
| 1 | chr8 | 68,8 | Gain | PT+Met+DCCs | 100 | 100 | 8q13.1-24.23 |
| 2 | chr17 | 14,3 | Gain | PT+Met+DCCs | 100 | 100 | 17q22-25.1 |
| 3 | chr3 | 34,9 | Loss | PT+Met+DCCs | 90 | 87,5 | 3p21.31-p12.2 |
| 4 | chr6 | 95,6 | Loss | PT+Met+DCCs | 100 | 100 | 6q13-27 |
| 5 | chr12 | 25,3 | Loss | PT+Met+DCCs | 100 | 100 | 12p13.33-11.23 |
| 6 | chr13 | 27,4 | Loss | PT+Met+DCCs | 100 | 100 | 13q14.11-21.33 |
| 7 | chr16 | 35,1 | Loss | PT+Met+DCCs | 90 | 87,5 | 16q23.2-24.2 |
| 8 | chr17 | 7,2 | Loss | PT+Met+DCCs | 90 | 87,5 | 17p13.1-11.2 |
| 9 | chr22 | 27,7 | Loss | PT+Met+DCCs | 90 | 87,5 | 22q11.21-13.31 |
| 10 | chr5 | 34,5 | Gain | DCCs | 70 | 87,5 | 5q32-35.3 |
| 11 | chr10 | 10,6 | Gain | DCCs | 10 | 12,5 | 10q26.11-26.3 |
| 12 | chr15 | 8,4 | Gain | DCCs | 30 | 37,5 | 15q26.1-26.3 |
| 13 | chrX | 17,4 | Gain | DCCs | 40 | 50 | Xq26.2-28 |
| 14 | chr1 | 35,1 | Loss | DCCs | 10 | 12,5 | 1p36.12-32.3 |
| 15 | chr4 | 10,2 | Loss | DCCs | 10 | 12,5 | 4q13.1-13.3 |
| 16 | chr4 | 14,8 | Loss | DCCs | 10 | 12,5 | 4q22.1-25 |
| 17 | chr9 | 14,4 | Loss | DCCs | 70 | 87,5 | 9q21.11-21.32 |
| 18 | chr10 | 1,9 | Loss | DCCs | 20 | 25 | 10p15.3-15.2 |
| 19 | chr10 | 20,3 | Loss | DCCs | 10 | 12,5 | 10p13-11.21 |
| 20 | chr11 | 25,9 | Loss | DCCs | 20 | 25 | 11p14.3-11.12 |
| 21 | chr12 | 2,4 | Loss | DCCs | 10 | 12,5 | 12q21.31-21.31 |
| 22 | chr15 | 14,0 | Loss | DCCs | 30 | 37,5 | 15q24.1-25.3 |
| 23 | chr17 | 6,9 | Loss | DCCs | 40 | 50 | 17q11.2-12 |
| 24 | chr18 | 4,1 | Loss | DCCs | 10 | 12,5 | 18p11.31-11.23 |
| 25 | chr19 | 20,8 | Loss | DCCs | 10 | 12,5 | 19p13.3-12 |
| 26 | chr21 | 3,6 | Loss | DCCs | 40 | 50 | 21q11.2-21.1 |
| 27 | chrX | 7,4 | Loss | DCCs | 20 | 25 | Xq11.1-13.1 |
| 28 | chr18 | 59,5 | Gain | PT | 10 | 0 | 18q11.1-23 |
| 29 | chr1 | 22,7 | Gain | PT+DCCs | 80 | 87,5 | 1q24.1-31.1 |
| 30 | chr1 | 10,0 | Gain | PT+DCCs | 80 | 87,5 | 1q41-42.12 |
| 31 | chr4 | 12,3 | Gain | PT+DCCs | 30 | 25 | 4q21.21-22.1 |
| 32 | chr8 | 8,6 | Gain | PT+DCCs | 20 | 12,5 | 8p23.3-23.1 |
| 33 | chr9 | 33,5 | Gain | PT+DCCs | 60 | 62,5 | 9p22.33-34.13 |
| 34 | chr11 | 44,3 | Loss | PT+DCCs | 80 | 87,5 | 11q14.3-q25 |
| 35 | chr20 | 11,9 | Gain | Met+DCCs | 80 | 87,5 | 20q13.13-13.33 |
| 36 | chr6 | 12,2 | Loss | Met+DCCs | 80 | 87,5 | 6p25.3-24.1 |
| 37 | chr8 | 26,4 | Loss | Met+DCCs | 80 | 87,5 | 8p22-11.21 |
| 38 | chr9 | 30,1 | Loss | Met+DCCs | 90 | 100 | 9p24.1-13.2 |
| 39 | chr10 | 7,2 | Loss | Met+DCCs | 50 | 50 | 10q21.1-21.2 |
| 40 | chr14 | 10,5 | Loss | Met+DCCs | 90 | 100 | 14q24.1-31.1 |
| 41 | chr18 | 41,7 | Loss | Met+DCCs | 80 | 87,5 | 18q12.2-23 |
| 42 | chr19 | 19,7 | Loss | Met+DCCs | 20 | 12,5 | 19q13.2-13.43 |
| 43 | chrX | 46,0 | Loss | Met+DCCs | 90 | 100 | Xp22.33-11.23 |

Table S3. Amount of aberrant intervals detected across the samples included in the case report study of an advanced breast cancer patient.

| **Sample ID** | **Count of aberrant genomic intervals** |
| --- | --- |
| Primary Tumor | 15 |
| Lymph Node Metastasis | 18 |
| Time Point 1: DCC #1 | 20 |
| Time Point 1: DCC #2 | 24 |
| Time Point 2: DCC #1 | 36 |
| Time Point 2: DCC #2 | 44 |
| Time Point 3: DCC #1 | 12 |
| Time Point 3: DCC #2 | 26 |
| Time Point 4: DCC #1 | 28 |
| Time Point 4: DCC #2 | 33 |
